# Revisiting the Association Between Calcium Channel Blockers and Parkinson's Disease in the E3N Cohort

**DOI:** 10.1002/mds.70336

**Published:** 2026-04-29

**Authors:** Emilie Moutard, Agnès Fournier, Émeline Courtois, Fanny Artaud, Pascale Tubert‐Bitter, Emmanuel Roze, Ismaïl Ahmed, Anne C.M. Thiébaut, Alexis Elbaz, Thi Thu Ha Nguyen

**Affiliations:** ^1^ Inserm, Gustave Roussy, Centre de recherche en épidémiologie et santé des populations (CESP) Université Paris‐Saclay (UVSQ) Villejuif France; ^2^ Inserm, Centre de recherche en épidémiologie et santé des populations (CESP) Université Paris‐Saclay (UVSQ) Villejuif France; ^3^ AP‐HP, Hôpital Pitié‐Salpêtrière, Département de Neurologie Sorbonne Université, Paris, France; INSERM U1127, CNRS 7225, Institut du Cerveau Paris France

**Keywords:** Parkinson's disease, calcium channel blockers, epidemiology, cohort studies

## Abstract

**Background:**

Previous studies on calcium channel blockers (CCBs) and Parkinson's disease (PD) risk reached conflicting conclusions.

**Objectives:**

We examined the relationship between CCBs and PD in the E3N cohort of French women followed for 15 years (2004–2018), while taking into account the potential for reverse causation.

**Methods:**

New users of CCBs were identified using drug reimbursement databases. We validated incident PD using several data sources. We described trajectories of CCBs use using mixed logistic models within a nested case–control study. We used Cox proportional hazards models for time‐varying variables to estimate the association between CCBs (overall, amlodipine, non‐amlodipine dihydropyridines [DiCCBs], non‐dihydropyridines [non‐DiCCBs]) and PD. Main analyses used a 5‐year lag; sensitivity analyses without a lag were performed for comparison.

**Results:**

Among 82,605 women, 603 developed PD and 13,022 used CCBs. Women who started non‐DiCCBs had higher PD risk than never users (hazard ratio [HR] = 1.56; 95% confidence interval [95% CI] = 0.99–2.46; *P*‐trend ≤ 0.02). Among non‐DiCCBs, diltiazem was associated with PD (HR = 2.20 [95% CI = 1.19–4.04]). There were no significant associations for CCBs overall, amlodipine, and non‐amlodipine DiCCBs. In analyses without a lag, amlodipine (HR = 1.45 [95% CI = 1.13–1.87]) and diltiazem (HR = 1.63 [95% CI = 1.02–2.60]) were associated with PD. Results are consistent with trajectories of CCBs prescriptions in PD patients and controls.

**Discussion:**

Overall, CCBs were not associated with PD but women who started using diltiazem had higher PD incidence in lagged analyses. Diltiazem and amlodipine were associated with PD in analyses without a lag. These findings are consistent with case reports of parkinsonism induced by specific CCBs and warrant further studies and surveillance. © 2026 The Author(s). *Movement Disorders* published by Wiley Periodicals LLC on behalf of International Parkinson and Movement Disorder Society.

Current treatments for Parkinson's disease (PD) fail to delay disease progression.^1^ Repurposing existing drugs may provide new treatment options. Dopaminergic neurons display L‐type calcium channels^2^; calcium plays a key role in neuronal function and is involved in cellular processes leading to cell death.^3^ Therefore, calcium channel blockers (CCBs), whose main indications are hypertension, coronary heart disease, and subarachnoid hemorrhage, have been considered as potential targets for PD.^3^

Dihydropyridine CCBs (DiCCBs: amlodipine, nimodipine, nifedipine, felodipine, lercanidipine, nicardipine, isradipine, lacidipine, nitrendipine, manidipine) are selective for L‐type calcium channels in vascular smooth muscles, while less selective non‐DiCCBs (bepridil, verapamil, diltiazem) also have cardiac effects. All CCBs are highly lipophilic and can penetrate the brain, especially during chronic treatment^4^, ^5^, ^6^; amlodipine crosses the blood–brain barrier less readily than other DiCCBs.^7^

Observational studies that investigated whether CCBs reduce PD risk reached inconsistent findings.^8^, ^9^, ^10^, ^11^, ^12^, ^13^, ^14^, ^15^ The latest meta‐analysis (three case–control, four cohort studies) showed that CCBs were associated with reduced PD risk (relative risk [RR] = 0.82, 95% confidence interval [95% CI] = 0.71–0.93, *P* = 0.016) with moderate heterogeneity (*I* ^2^ = 55.7%, *P* = 0.027).^16^ The association was slightly stronger for cohort (RR = 0.74; 95% CI = 0.65–0.83) than case–control studies (RR = 0.83; 95% CI = 0.67–1.00), and DiCCBs (RR = 0.70; 95% CI = 0.66–0.89) than non‐DiCCBs (RR = 0.78; 95% CI = 0.58–0.83).^16^

Previous results must be interpreted in the light of some limitations (eg, diagnostic misclassification in medico‐administrative databases; baseline exposure not updated over the follow‐up; residual confounding). In addition, reverse causation represents an important issue because prodromal symptoms (eg, hypotension, constipation) may influence CCBs use.^17^ Studies with a long follow‐up are needed to address this issue.

We studied the relationship between CCBs and PD in a cohort of French women followed for 15 years, while taking into account the methodological limitations mentioned earlier, in particular the risk of reverse causation.

## Subjects and Methods

### Participants

E3N is a French cohort study of 98,995 women, born between 1925 and 1950, recruited in 1990, and affiliated with a French national health insurance plan covering mostly teachers (Mutuelle Générale de l'Education Nationale [MGEN]).^18^

Participants (~20% of invited women) completed a self‐administered questionnaire on lifestyle and medical history at baseline (1990) and every 24–36 months thereafter. Eleven waves are available (last, Q11‐2014; average response rate at each questionnaire = 80%). Only 3% of E3N participants are lost to follow‐up.^18^ Drug and medical consultation claims data were available between January 1, 2004 and December 31, 2018. Causes of death are available until December 31, 2014.

All participants signed informed consent, in compliance with the rules of the French National Commission for Data Protection and Privacy which approved the study. The study protocol is registered at clinicaltrials.gov (NCT03285230).

### Calcium Channel Blockers

We used drug claims databases to assess CCBs use (January 1, 2004–December 31, 2018). These databases include detailed information on drugs reimbursed, date of purchase, number of tablets delivered, and strength of tablets. Anatomical Therapeutic‐Chemical (ATC) codes were used to identify CCBs (second‐level ATC‐C08; Table S1).

We constructed treatment episodes based on purchase dates and duration of use. Duration of use was obtained for each claim by dividing dose (number of tablets × tablet strength) by a hypothetical daily dose (minimum daily dose for the main indication of each CCB according to French prescription policies). To account for differences in recommended daily doses for different CCBs, doses were converted into defined daily doses (DDDs).^19^ We computed the cumulative duration and cumulative dose each month by summing durations and doses. The average daily dose was computed as cumulative dose divided by cumulative duration.

We first considered time‐varying ever use of any CCB. We then distinguished DiCCBs from non‐DiCCBs because they have different chemical structures and properties, and because previous studies reported findings for these two groups and tended to show stronger associations for DiCCBs. Among DiCCBs, we distinguished amlodipine from other DiCCBs given differences in its transportation through the blood–brain barrier.^7^ In exploratory analyses, we evaluated the most frequent molecules (at least 10 exposed cases; DiCCBs: amlodipine, lercanidipine; non‐DiCCBs: verapamil, diltiazem; Table S1).

### Parkinson's Disease

Our approach to ascertain PD is described in detail elsewhere.^20^ Potential PD patients were identified through self‐reported doctor‐diagnoses of PD, antiparkinsonian drug claims (ATC‐N04), and death certificates, and were contacted to confirm the diagnosis. For women who confirmed a PD/parkinsonism diagnosis and those who could not be contacted, we obtained detailed medical records from their neurologists; they were reviewed by an expert panel to adjudicate PD status (definite, probable, possible, no PD).^21^ Definite PD was defined as at least two of four cardinal signs (rest tremor, rigidity, bradykinesia, impaired postural reflexes), with a positive and significant response to treatment, without early signs of more extensive nervous system involvement and drug‐induced parkinsonism.^21^ When the same criteria were fulfilled with insufficient information regarding treatment response, we attributed a diagnosis of probable PD. Women were included in the possible PD group when the clinical description was suggestive, but there was insufficient information to assign a definite/probable diagnosis. We retained those with definite/probable PD for the analyses.

When no medical records were available, we predicted PD status using an algorithm based on antiparkinsonian drug claims and medical visits that was previously validated against a clinical diagnosis (94% sensitivity and 88% specificity among women who self‐reported PD or used antiparkinsonian drugs).^20^ The proportions of PD diagnoses based on medical records and the algorithm are 62% and 38%, respectively.

Date of PD diagnosis (month/year) was the date when antiparkinsonian drugs were started if PD was diagnosed the same year; if PD diagnosis preceded that date, we used June of the year of diagnosis.

We showed comparable PD incidence rates in E3N and Western European women (Global Burden of Disease) over the same years (1992–2018), supporting the validity of our approach.^20^

### Covariates

A detailed description is available in the Supplementary Methods. Analyses were adjusted for covariates associated with PD risk (rural residence,^22^ education,^23^ age at menarche,^24^ parity,^24^ artificial menopause,^24^ physical activity,^25^ caffeine intake,^22^ smoking,^22^ body mass index (BMI),^26^ gout,^27^ and type 2 diabetes^28^). Use of medical services was taken into account by adjusting for the time‐varying number of medical consultations in the last 6 months.^29^ Analyses were adjusted for main CCBs indications (hypertension, cardiovascular disease [CVD]) and drugs inversely associated with PD in E3N (lipophilic statins,^30^ long/ultra‐long‐acting β2‐agonists^31^).

### Study Population

Figure S1 illustrates participants' selection for survival analyses. We implemented a new‐user design by defining a 6‐month washout period to include only incident CCBs users.^32^ Thus, evaluation of exposure to CCBs commenced on July 1, 2004.

To address reverse causation, we included a 5‐year lag.^33^ PD follow‐up started 5 years after CCBs exposure assessment started (July 1, 2009). We chose a 5‐year lag because we previously showed that the number of medical consultations increased in cases compared with controls within 5 years before diagnosis; more frequent medical contacts within this period could lead to more frequent changes in prescriptions in patients compared with controls.^30^ In addition, one study showed that hypotension was more frequent in PD patients than controls in the 5 years before diagnosis but not 5–10 years.^34^ Time‐varying covariates were lagged in the same way as CCBs.

We retained in our analyses women without PD as of July 1, 2009, and with information on drug reimbursements thereafter. End of follow‐up was the earliest of PD diagnosis date, death, end of any drug reimbursements +5 years (for a small proportion of women who left the MGEN insurance scheme) or December 31, 2018.

### Statistical Analysis

Statistical analyses were carried out using SAS 9.4 (SAS Institute Inc., Cary, NC, USA) and Stata 16 (StataCorp, College Station, TX, USA). Two‐tailed *P*‐values ≤0.05 were considered statistically significant.

All analyses are reported transparently with effect sizes, confidence intervals, and *P*‐values; given that exposures were prespecified, limited in number, and not independent, no correction for multiple comparisons was applied.^35^

Baseline participants' characteristics were described overall and according to CCBs use over the follow‐up and PD status at the end of follow‐up.

Two complementary sets of analyses were performed.

### Trajectories of CCBs Use

We set up a nested case–control study to describe prescriptions' trajectories in the years before diagnosis (overall, amlodipine, non‐amlodipine DiCCBs, non‐DiCCBs) in PD patients compared with controls. PD cases were matched to 20 controls each using incidence density sampling.^36^ Controls were PD‐free and matched to cases by age at diagnosis year (index date, Y0).

Trajectories of the frequency of ≥1 annual prescription of CCBs prior to Y0 were estimated using mixed‐effects logistic regression with a backward timescale going back from Y0 to the beginning of the study and random intercepts and slopes (unstructured variance). Time was modeled using polynomials. As the number of participants exposed to CCBs at the beginning of follow‐up was very low (new‐user design), trajectories were estimated over 10 years before Y0. The model was adjusted for age at Y0. Annual mean differences between cases and controls were statistically significant when 95% CIs did not include 0.

### CCBs and PD Incidence

To estimate associations of CCBs with PD incidence, we used Cox proportional hazards regression for time‐varying variables with age as the time scale. Associations were estimated through hazard ratios (HRs). Model 1 (M1) was unadjusted. M2 was further adjusted for variables associated with PD in the literature (M1 + urban/rural residence, education, parity, age at menarche, caffeine consumption, smoking, BMI, physical activity, menopausal status, number of medical consultations in the last 6 months, diabetes, gout). M3 was further adjusted for CCBs indications and other medications associated with PD (M2 + CVD, hypertension, lipophilic statins, long/ultra‐long‐acting β2‐agonists). Given the small proportion of missing values, they were coded as a specific category.

Time‐varying exposure to CCBs included: ever use (any CCBs, amlodipine, non‐amlodipine DiCCBs, non‐DiCCBs, individual molecules), cumulative dose, cumulative duration, and average daily dose. Models including different subtypes/molecules were mutually adjusted (correlated exposures). We performed exploratory analyses for ever use of individual molecules including the four most frequent drugs (amlodipine, lercanidipine, verapamil, diltiazem) and a single group combining all other CCBs.

Continuous exposure variables were categorized into three or four groups depending on the number of exposed cases: never use (reference) and exposed groups based on the median or tertiles of distributions among users. To test for linear trend, we defined ordinal variables based on the median of continuous variables in each group.

### Sensitivity Analyses

We performed several sensitivity analyses (described in more detail in the Supplementary Material):

- With a 7‐year lag and without a lag;
- Including prevalent CCBs users (5‐year lag);
- Adjusted for uncommon CCBs indications (cardiac arrhythmia, constipation);
- With a more specific version of the algorithm (specificity = sensitivity = 90%).

### Statistical Power

The statistical power to detect HRs≤0.74 (based on the meta‐analysis of cohort studies)^16^ was 80% for analyses with a 5‐year lag and 96% for analyses without a lag.

## Results

### Participants' Characteristics

Table 1 shows participants' characteristics, overall and according to ever use of CCBs. The study population comprised 82,605 women (mean age = 62.7 years, standard deviation [SD] = 6.3); 13,022 (15.8%) women started using CCBs. Compared with never users, ever users were older and less active, and had lower education, earlier menarche, more children, higher BMI, and more frequent medical contacts and comorbidities, including hypertension, CVD, and cardiac arrhythmia. They were more often postmenopausal, and lipophilic statins or long/ultra‐long‐acting β2‐agonist users. They consumed less caffeine and were less often current smokers.

**TABLE 1:** Baseline participants' characteristics, overall and by calcium channel blockers exposure at the end of the follow‐up

Characteristics in 2004		All women (N = 82,605) n (%)	CCBs use at the end of follow‐up	
			Ever users (N = 13,022) n (%)	Never users (N = 69,583) n (%)
Mean (SD) age (years)		62.7 (6.3)	65.2 (6.6)	62.2 (6.1)
Residence	Rural	11,160 (14.8)	1677 (14.1)	9483 (14.9)
	Urban	64,225 (85.2)	10,235 (85.9)	53,990 (85.1)
	Missing data	7220 (8.7)	1110 (8.5)	6110 (8.8)
Education	<High school	9069 (11.5)	1759 (14.1)	7310 (10.9)
	≤2 university years	40,369 (51.0)	6648 (53.4)	33,721 (50.6)
	>2 university years	29,698 (37.5)	4044 (32.5)	25,654 (38.5)
	Missing data	3469 (4.2)	571 (4.4)	2898 (4.2)
Parity	Nulliparous	10,014 (12.2)	1500 (11.6)	8514 (12.3)
	1 child	13,530 (16.5)	2053 (15.9)	11,477 (16.6)
	2 children	35,592 (43.4)	5282 (41.0)	30,310 (43.9)
	≥3 children	22,854 (27.9)	4067 (31.5)	18,787 (27.2)
	Missing data	615 (0.7)	120 (0.9)	495 (0.7)
Age at menarche (years)	<12	16,903 (21.0)	2713 (21.3)	14,190 (20.9)
	12	20,465 (25.4)	3217 (25.3)	17,248 (25.4)
	≥13	43,258 (53.6)	6788 (53.4)	36,470 (53.7)
	Missing data	1979 (2.4)	304 (2.3)	1675 (2.4)
Caffeine consumption (mg/day)	<94	15,739 (24.9)	2414 (24.7)	13,325 (24.9)
	94–173	15,638 (24.7)	2461 (25.3)	13,177 (24.6)
	174–270	15,831 (25.0)	2459 (25.3)	13,372 (25.0)
	≥271	16,075 (25.4)	2401 (24.7)	13,674 (25.5)
	Missing data	19,322 (23.4)	3287 (25.2)	16,035 (23.0)
Smoking status	Non‐smoker	43,788 (53.0)	7224 (55.5)	36,564 (52.6)
	Former smoker	29,542 (35.8)	4469 (34.3)	25,073 (36.0)
	Current smoker	9275 (11.2)	1329 (10.2)	7946 (11.4)
Body mass index (kg/m^2^)	<18.5	2610 (3.1)	341 (2.6)	2269 (3.3)
	18.5–24.9	55,351 (67.1)	7426 (57.1)	47,925 (68.9)
	25.0–29.9	19,224 (23.3)	3799 (29.2)	15,425 (22.2)
	≥30.0	5336 (6.5)	1438 (11.1)	3898 (5.6)
	Missing data	84 (0.1)	18 (0.1)	66 (0.1)
Physical activity	Quartile1	20,620 (25.0)	3575 (27.5)	17,045 (24.5)
	Quartile2	20,620 (25.0)	3190 (24.5)	17,430 (25.1)
	Quartile3	20,620 (25.0)	3048 (23.5)	17,572 (25.3)
	Quartile4	20,620 (25.0)	3183 (24.5)	17,437 (25.1)
	Missing data	125 (0.2)	26 (0.2)	99 (0.1)
Menopause	No	1314 (1.6)	127 (1.0)	1187 (1.8)
	Natural	67,406 (84.8)	10,492 (83.2)	56,914 (85.1)
	Artificial	7674 (9.7)	1531 (12.2)	6143 (9.2)
	Unknown type	3088 (3.9)	459 (3.6)	2629 (3.9)
	Missing data	3123 (3.8)	413 (3.2)	2710 (3.9)
Number of consultations^a^	<2	13,032 (15.8)	1361 (10.5)	11,671 (16.8)
	2–3	23,094 (27.9)	3199 (24.6)	19,895 (28.6)
	4–6	25,674 (31.1)	4380 (33.6)	21,294 (30.6)
	≥7	20,805 (25.2)	4082 (31.3)	16,723 (24.0)
Comorbidities	Type 2 diabetes	2284 (2.8)	757 (5.8)	1527 (2.2)
	Hypertension	21,692 (26.3)	6770 (52.0)	14,922 (21.4)
	Cardiovascular disease	1699 (2.1)	504 (3.9)	1195 (1.7)
	Gout	1180 (1.4)	362 (2.8)	818 (1.2)
	Constipation	18,037 (21.8)	2771 (21.3)	15,266 (21.9)
	Cardiac arrhythmia	3751 (5.9)	938 (9.7)	2813 (5.3)
	Missing data	19,302 (23.4)	3297 (25.3)	16,005 (23.0)
Comedications	Lipophilic statins	9057 (11.0)	1978 (15.2)	7079 (10.2)
	β2‐agonists (LABA, ultra‐LABA)	3128 (3.8)	702 (5.4)	2426 (3.5)

Abbreviations: CCB, calcium channels blocker; SD, standard deviation; LABA, long‐acting.

^a^ Evaluated during the first 6 months of 2004.

Between July 1, 2004 and December 31, 2018, 806 women developed PD at a mean age of 75.1 years (SD = 6.2); the source of patients is described in Table S2. After excluding those who developed PD during the first 5 years, analyses are based on 603 participants. Compared with those who did not, women who developed PD were older, less often obese, more frequently postmenopausal, and had more children and frequent hypertension, CVD, and constipation (Table S3). They had lower physical activity, consumed less caffeine, and were less often current smokers.

### Trajectories of CCBs

**FIG. 1:**
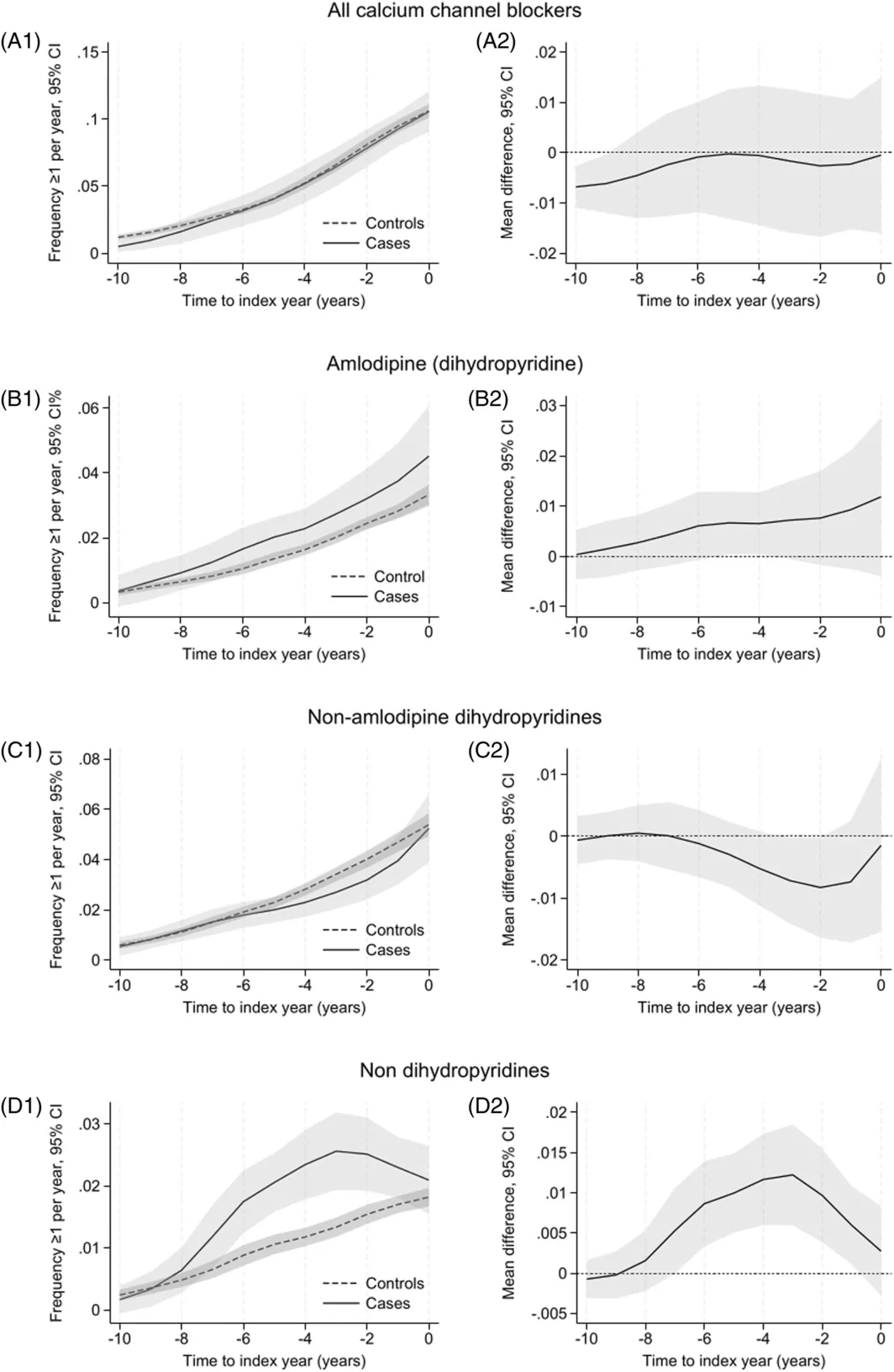
Trajectories of calcium channel blockers (CCBs) prescriptions (any type, amlodipine, non‐amlodipine dihydropyridines, and non‐dihydropyridines) in cases and controls before the date of Parkinson's disease (PD) diagnosis (year 0). The nested case–control study included 806 incident PD patients and 16,027 controls; 792 cases were matched to 20 controls; 11 cases to 10–19 controls and 3 cases to <10 controls. The figures show estimates (95% confidence intervals, CIs) of the frequency of ≥1 annual prescriptions of (A1) all CCBs, (B1) amlodipine, (C1) non‐amlodipine dihydropyridines, and (D1) non‐dihydropyridines in cases (solid line) and controls (dashed line) aged 75 years at the index date (Y0 = 0), based on marginal predictions of a mixed logistic regression model with time in years (backward scale) coded as polynomials. Plots A2, B2, C2, and D2 show the corresponding average annual differences between cases and controls; differences whose CIs do not include 0 are statistically significant (*P* ≤ 0.05).

The frequency of ≥1 annual CCBs prescription increased similarly over time in cases and controls (Fig. 1‐A1/A2).

PD patients tended to be more often treated with amlodipine than controls (Fig. 1‐B1/B2), with a significant difference 6 years before diagnosis (0.7%, 95% CI = 0.4–1.3, *P* = 0.036), while the opposite was observed for non‐amlodipine DiCCBs (Fig. 1‐C1/C2).

The frequency of ≥1 annual prescription of non‐DiCCB was similar in cases and controls between 7 and 10 years before diagnosis; it increased more in cases compared with controls up to 1 year before diagnosis, then became similar again just before; 6 years before diagnosis, the frequency was 1% in controls versus 2% in cases (difference = 1%, 95% CI = 0.5–1.5, *P* < 0.001) (Fig. 1‐D1/D2).

### CCBs Use and PD Incidence

Table 2 summarizes analyses of the relationship between CCBs and PD. Compared with never users, ever users had a non‐significant higher PD incidence (HR = 1.14, 95% CI = 0.87–1.50). There was no dose–response relationship for any exposure variable.

**TABLE 2:** Association of calcium channel blockers use with the incidence of Parkinson's disease: analyses with a 5‐year exposure lag

CCBs	Cases (N = 603) n	IR	Model 1 HR (95% CI)^a^	*P*‐value	Model 2 HR (95% CI)^b^	*P*‐value	Model 3 HR (95% CI)^c^	*P*‐value
Never users	538	0.78 (0.71–0.85)	1.00 (Reference)	0.43	1.00 (Reference)	0.37	1.00 (Reference)	0.34
Ever users	65	1.12 (0.88–1.43)	1.11 (0.86–1.44)		1.13 (0.87–1.47)		1.14 (0.87–1.50)	
Cumulative dose (DDDs)								
Never	538	0.78 (0.71–0.85)	1.00 (Reference)	0.49^d^	1.00 (Reference)	0.46^d^	1.00 (Reference)	0.45^d^
<64	19	0.98 (0.63–1.54)	1.02 (0.64–1.61)		1.04 (0.66–1.65)		1.05 (0.66–1.67)	
64–505	23	1.19 (0.79–1.79)	1.18 (0.77–1.79)		1.19 (0.78–1.82)		1.20 (0.78–1.85)	
≥506	23	1.19 (0.79–1.79)	1.14 (0.75–1.73)		1.15 (0.75–1.76)		1.17 (0.76–1.80)	
Cumulative duration (days)								
Never	538	0.78 (0.71–0.85)	1.00 (Reference)	0.29^d^	1.00 (Reference)	0.27^d^	1.00 (Reference)	0.26^d^
<60	18	0.98 (0.62–1.55)	1.02 (0.63–1.63)		1.04 (0.65–1.66)		1.05 (0.65–1.69)	
60–486	22	1.08 (0.71–1.64)	1.07 (0.70–1.65)		1.09 (0.71–1.68)		1.10 (0.71–1.71)	
≥487	25	1.29 (0.87–1.91)	1.24 (0.82–1.85)		1.25 (0.83–1.88)		1.27 (0.84–1.92)	
Average daily dose (DDDs)								
Never	538	0.78 (0.71–0.85)	1.00 (Reference)	0.56^d^	1.00 (Reference)	0.50^d^	1.00 (Reference)	0.47^d^
<1	14	1.47 (0.87–2.49)	1.49 (0.88–2.54)		1.51 (0.88–2.57)		1.54 (0.90–2.63)	
=1	37	1.10 (0.80–1.52)	1.10 (0.79–1.54)		1.11 (0.79–1.56)		1.12 (0.79–1.58)	
>1	14	0.93 (0.55–1.57)	0.90 (0.53–1.54)		0.93 (0.54–1.58)		0.94 (0.54–1.61)	

Abbreviations: CCB, calcium channel blocker; IR, incidence rate (per 1000 person‐years); HR, hazard ratio; CI, confidence interval; DDD, defined daily dose.

^a^ HRs, 95% CIs, and *P*‐values were computed using Cox proportional hazards regression for time‐varying variables with age as the timescale.

^b^ Model 1 further adjusted for time‐invariant variables (type of residence, education level, parity, age at first menarche, and caffeine consumption) and time‐varying variables (smoking status, body mass index, physical activity level, menopausal status, number of consultations in the previous 6 months, type 2 diabetes, and gout).

^c^ Model 2 further adjusted for time‐varying cardiovascular diseases, hypertension, lipophilic statins, and β2‐agonists (long‐acting and ultra‐long‐acting).

^d^ *P*‐value for linear trend.

During the follow‐up, 5139 (6.2%) women took amlodipine, 7878 (9.5%) non‐amlodipine DiCCBs, and 2763 (3.3%) non‐DiCCBs. Ever users of non‐DiCCBs had higher PD incidence compared with non‐users (HR = 1.56, 95% CI = 0.99–2.46; Table 3). HRs increased significantly with increasing cumulative dose (*P* = 0.02), with a two‐fold higher risk for those above the median (HR = 2.01, 95% CI = 1.15–3.51). A dose–response relationship was also present for cumulative duration (*P* = 0.01), with a similar significant association for duration>median (HR = 2.01, 95% CI = 1.15–3.52). The trend for average daily dose was borderline significant (*P* = 0.05) with no difference between those below or above the median. There were no significant associations for amlodipine and other DiCCBs (Table 3).

**TABLE 3:** Association of amlodipine, non‐amlodipine dihydropyridine calcium channel blockers (DiCCBs), and non‐DiCCBs use with the incidence of Parkinson's disease: analyses with a 5‐year exposure lag

CCBs	Cases (N = 603) n	IR	Model 1 HR (95% CI)^a^	*P*‐value	Model 2 HR (95% CI)^b^	*P*‐value	Model 3 HR (95% CI)^c^	*P*‐value
Amlodipine								
Never users	581	0.80 (0.73–0.86)	1.00 (Reference)	0.84	1.00 (Reference)	0.79	1.00 (Reference)	0.75
Ever users	22	1.13 (0.74–1.71)	1.05 (0.67–1.62)		1.06 (0.68–1.65)		1.08 (0.69–1.68)	
Cumulative dose (DDDs)								
Never	581	0.80 (0.73–0.86)	1.00 (Reference)	0.32^d^	1.00 (Reference)	0.30^d^	1.00 (Reference)	0.27^d^
≤146	8	0.82 (0.41–1.64)	0.77 (0.38–1.56)		0.78 (0.38–1.58)		0.79 (0.39–1.61)	
>146	14	1.44 (0.85–2.43)	1.32 (0.77–2.26)		1.34 (0.78–2.30)		1.36 (0.79–2.35)	
Cumulative duration (days)								
Never	581	0.80 (0.73–0.86)	1.00 (Reference)	0.30^d^	1.00 (Reference)	0.28^d^	1.00 (Reference)	0.26^d^
≤134	8	0.82 (0.41–1.64)	0.77 (0.38–1.56)		0.78 (0.39–1.59)		0.79 (0.39–1.61)	
>134	14	1.44 (0.85–2.43)	1.32 (0.77–2.26)		1.34 (0.78–2.30)		1.36 (0.79–2.34)	
Average daily dose (DDDs)								
Never	581	0.80 (0.73–0.86)	1.00 (Reference)	0.87^d^	1.00 (Reference)	0.92^d^	1.00 (Reference)	0.96^d^
≤1	21	1.34 (0.87–2.05)	1.24 (0.79–1.94)		1.26 (0.80–1.97)		1.27 (0.81–2.00)	
>1	1	0.26 (0.04–1.87)	0.24 (0.03–1.72)		0.25 (0.03–1.77)		0.25 (0.04–1.80)	
Non‐amlodipine DiCCBs								
Never users	566	0.79 (0.73–0.86)	1.00 (Reference)	0.96	1.00 (Reference)	0.90	1.00 (Reference)	0.91
Ever users	37	1.08 (0.78–1.48)	1.01 (0.72–1.42)		1.02 (0.72–1.44)		1.02 (0.72–1.45)	
Cumulative dose (DDDs)								
Never	566	0.79 (0.73–0.86)	1.00 (Reference)	0.98^d^	1.00 (Reference)	0.94^d^	1.00 (Reference)	0.94^d^
≤162	18	1.05 (0.66–1.66)	1.00 (0.62–1.62)		1.02 (0.63–1.65)		1.02 (0.63–1.65)	
>162	19	1.10 (0.70–1.73)	1.02 (0.64–1.63)		1.03 (0.65–1.65)		1.03 (0.64–1.65)	
Cumulative duration (days)								
Never	566	0.79 (0.73–0.86)	1.00 (Reference)	0.85^d^	1.00 (Reference)	0.88^d^	1.00 (Reference)	0.87^d^
≤150	19	1.10 (0.70–1.73)	1.06 (0.66–1.69)		1.08 (0.68–1.72)		1.08 (0.67–1.73)	
>150	18	1.05 (0.66–1.66)	0.97 (0.60–1.56)		0.98 (0.61–1.58)		0.98 (0.60–1.59)	
Average daily dose (DDDs)								
Never	566	0.79 (0.73–0.86)	1.00 (Reference)	0.92^d^	1.00 (Reference)	0.86^d^	1.00 (Reference)	0.87^d^
≤1	26	1.08 (0.74–1.59)	1.02 (0.68–1.52)		1.03 (0.68–1.54)		1.02 (0.68–1.54)	
>1	11	1.06 (0.59–1.92)	1.00 (0.55–1.82)		1.02 (0.55–1.86)		1.02 (0.56–1.88)	
Non‐DiCCBs								
Never users	583	0.79 (0.73–0.86)	1.00 (Reference)	0.08	1.00 (Reference)	0.08	1.00 (Reference)	0.06
Ever users	20	1.46 (0.94–2.26)	1.49 (0.95–2.34)		1.50 (0.96–2.36)		1.56 (0.99–2.46)	
Cumulative dose (DDDs)								
Never	583	0.79 (0.73–0.86)	1.00 (Reference)	0.02^d^	1.00 (Reference)	0.02^d^	1.00 (Reference)	0.02^d^
≤94.5	7	1.02 (0.49–2.15)	1.07 (0.51–2.26)		1.08 (0.51–2.29)		1.12 (0.53–2.37)	
>94.5	13	1.90 (1.10–3.27)	1.92 (1.10–3.34)		1.92 (1.10–3.35)		2.01 (1.15–3.51)	
Cumulative duration (days)								
Never	583	0.79 (0.73–0.86)	1.00 (Reference)	0.02^d^	1.00 (Reference)	0.02^d^	1.00 (Reference)	0.01^d^
≤106	7	1.02 (0.49–2.14)	1.06 (0.50–2.25)		1.07 (0.51–2.27)		1.11 (0.52–2.35)	
>106	13	1.90 (1.10–3.28)	1.92 (1.11–3.34)		1.93 (1.11–3.36)		2.01 (1.15–3.52)	
Average daily dose (DDDs)								
Never	583	0.79 (0.73–0.86)	1.00 (Reference)	0.08^d^	1.00 (Reference)	0.07^d^	1.00 (Reference)	0.05^d^
≤1	8	1.47 (0.73–2.94)	1.50 (0.74–3.02)		1.50 (0.74–3.02)		1.56 (0.77–3.15)	
>1	12	1.46 (0.83–2.56)	1.48 (0.83–2.64)		1.50 (0.84–2.67)		1.55 (0.86–2.76)	

Abbreviations: CCB, calcium channel blocker; IR, incidence rate (per 1000 person‐years); HR, hazard ratio; CI, confidence interval; DDD, defined daily dose; DiCCB, dihydropyridine calcium channel blocker.

^a^ HRs, 95% CIs, and *P*‐values were computed using Cox proportional hazards regression for time‐varying variables with age as the timescale. Amlodipine, the other non‐amlodpine DiCCBS (felodipine, isradipine, lacidipine, manidipine, nicardipine, nifedipine, nimodipine, and lercanidipine), and non‐DiCCBs (bepridil, verapamil, and ditiazem) were included in the same mutually adjusted model.

^b^ Model 1 further adjusted for time‐invariant variables (type of residence, education level, parity, age at first menarche, and caffeine consumption) and for time‐varying variable (smoking status, body mass index, physical activity level, menopausal status, number of consultations in the previous 6 months, type 2 diabetes, and gout).

^c^ Model 2 further adjusted for time‐varying cardiovascular diseases, hypertension, lipophilic statins, and β2‐agonists (long‐acting and ultra‐long‐acting).

^d^ *P*‐values for linear trend.

Ever diltiazem users had higher PD incidence than never users (HR = 2.20, 95% CI = 1.19–4.04; Table 4). There were no significant association for amlodipine, lercanidipine, verapamil, or other CCBs.

### Sensitivity Analyses

Analysis with a 7‐year lag (Table S4): compared with main analyses, the association between non‐DiCCBs and PD decreased (HR = 1.26, 95% CI = 0.67–2.37) while diltiazem remained associated with a similar HR (HR = 2.44, 95% CI = 1.14–5.21).

**TABLE 4:** Association of amlodipine, lercanidipine, verapamil, diltiazem, and other calcium channel blockers use with the incidence of Parkinson's disease: analyses with a 5‐year exposure lag

CCBs	Cases (N = 603) n	IR	Model 1 HR (95% CI)^a^	*P*‐value	Model 2 HR (95% CI)^b^	*P*‐value	Model 3 HR (95% CI)^c^	*P*‐value
Amlodipine								
Never users	581	0.80 (0.73–0.86)	1.00 (Reference)	0.86	1.00 (Reference)	0.80	1.00 (Reference)	0.76
Ever users	22	1.13 (0.74–1.71)	1.04 (0.67–1.61)		1.06 (0.68–1.64)		1.07 (0.69–1.67)	
Lercanidipine								
Never users	575	0.79 (0.73–0.86)	1.00 (Reference)	0.23	1.00 (Reference)	0.22	1.00 (Reference)	0.23
Ever users	28	1.30 (0.90–1.89)	1.27 (0.86–1.88)		1.28 (0.86–1.90)		1.28 (0.86–1.90)	
Verapamil								
Never users	592	0.80 (0.74–0.87)	1.00 (Reference)	0.65	1.00 (Reference)	0.63	1.00 (Reference)	0.57
Ever users	11	1.21 (0.67–2.18)	1.15 (0.63–2.11)		1.16 (0.63–2.12)		1.19 (0.65–2.19)	
Diltiazem								
Never users	592	0.79 (0.73–0.86)	1.00 (Reference)	0.01	1.00 (Reference)	0.02	1.00 (Reference)	0.01
Ever users	11	2.13 (1.18–3.85)	2.12 (1.16–3.88)		2.11 (1.15–3.86)		2.20 (1.19–4.04)	
Other CCBs^d^								
Never users	591	0.81 (0.74–0.87)	1.00 (Reference)	0.17	1.00 (Reference)	0.18	1.00 (Reference)	0.19
Ever users	12	0.75 (0.43–1.32)	0.66 (0.37–1.19)		0.67 (0.38–1.21)		0.67 (0.38–1.21)	

Abbreviations: CCB, calcium channel blocker; IR, incidence rate (per 1000 person‐years); HR, hazard ratio; CI, confidence interval; DDD, defined daily dose.

^a^ HRs, 95% CIs, and *P*‐values were computed using Cox proportional hazards regression for time‐varying variables with age as the timescale. Amlodipine, lercanidipine, verapamil, ditiazem, and other calcium channel blockers (felodipine, isradipine, lacidipine, manidipine, nicardipine, nifedipine, nimodipine, bepridil) were included in the same mutually adjusted model.

^b^ Model 1 further adjusted for time‐invariant variables (type of residence, education level, parity, age at first menarche, and caffeine consumption) and for time‐varying variables (smoking status, body mass index, physical activity level, menopausal status, number of consultations in the previous 6 months, type 2 diabetes, and gout).

^c^ Model 2 further adjusted for time‐varying cardiovascular diseases, hypertension, lipophilic statins and β2‐agonists (long‐acting and ultra‐long‐acting).

^d^ Felodipine, isradipine, lacidipine, manidipine, nicardipine, nifedipine, nimodipine, bepridil.

Analysis without a lag (Fig. S2, Table S5): for CCBs, PD risk tended to increase with cumulative dose (*P* = 0.07) and duration (*P* = 0.06; Table S6). The positive association became weaker and non‐significant for non‐DiCCBs, while it increased and became significant for amlodipine (HR = 1.44, 95% CI = 1.12–1.85), with a dose–response relationship for all exposure indices (Table S7). Among individual CCBs, there was a positive association for diltiazem (HR = 1.63, 95% CI = 1.02–2.60; Table S8).

Analyses for time since first/last use (Table S9): for amlodipine, PD incidence was higher in women who started using amlodipine <5 years before (HR_<2.5y_ = 1.47, 95% CI = 1.01–2.14; HR_2.5–4.9y_ = 1.66, 95% CI = 1.10–2.50), while the association was weaker and not significant for women who started treatment ≥5 years earlier (HR_≥5y_ = 1.23, 95% CI = 0.79–1.90). PD incidence was higher in women who were current amlodipine users or who had stopped <2.5 years before (HR = 1.50, 95% CI = 1.13–2.00), while HRs decreased and were non‐significant for women who had stopped for more than 2.5 years. For non‐DiCCBs, PD incidence increased with time since first use (P = 0.05); women who stopped taking non‐DiCCBs 2.5–4.9 years before had higher risk (HR = 1.89, 95% CI = 0.97–3.66).

Analyses including prevalent CCBs users (5‐year lag): results were generally similar compared with analyses based on incident users (Table S10). Associations were slightly weaker for non‐DiCCBs (HR = 1.35, 95% CI = 0.96–1.89) but remained statistically significant for diltiazem (HR = 1.75, 95% CI = 1.11–2.77).

Adjustment for uncommon CCBs indications (cardiac arrhythmia, constipation; Table S11): results were unchanged.

More specific version of the algorithm (Table S12): most associations tended to become stronger and more significant.

## Discussion

Women who started using CCBs did not have lower PD risk over a 15‐year follow‐up. Alternatively, non‐DiCCBs use (notably diltiazem) was associated with higher incidence in analyses with a 5‐year lag; diltiazem and amlodipine were associated with higher PD incidence in sensitivity analyses without a lag. Results are consistent with trajectories of non‐DiCCBs and amlodipine prescriptions in patients prior to diagnosis and controls.

Five cohort^10^, ^11^, ^13^, ^14^, ^15^ and three case–control^8^, ^9^, ^12^ studies investigated the association between CCBs and PD; five^9^, ^12^, ^13^, ^14^, ^15^ reported an inverse relationship and three^8^, ^10^, ^11^ reported no association (Tables S13 and S14). All previous cohort studies showing a significant inverse association were based on medico‐administrative databases.^13^, ^14^, ^15^ The validity of PD diagnoses derived from these databases is questionable^37^, ^38^; this issue is particularly relevant for CCBs since one of their main indications is hypertension, a risk factor for vascular parkinsonism. In addition, major confounders (eg, smoking, physical activity) are usually not available in these databases, leading to residual confounding.

Reverse causation represents an important issue for studies on CCBs and PD because prodromal symptoms could influence CCBs use prior to diagnosis.^17^ Hypotension is more frequent in PD patients than controls before diagnosis^34^; therefore, antihypertensive drugs could be prescribed less often to PD patients before diagnosis. In addition, in our study, the number of medical contacts increased in PD patients in the 5 years prior to diagnosis compared with controls^30^; this difference could lead to more frequent blood pressure measurements and changes in prescriptions in PD patients before diagnosis. Constipation, a frequent side effect of some CCBs (especially verapamil and nifedipine),^39^ could lead to avoidance of these medications in patients with prodromal constipation, also leading to an inverse association. The inclusion of a 5‐year lag and analyses of prescriptions trajectories represent important features of our study. Four previous studies implemented an exposure lag^8^, ^12^, ^13^, ^14^; two studies with a 1.5‐ and 5‐year lag found no association between CCBs and PD.^8^, ^14^ In one, a statistically significant inverse association in analyses without a lag disappeared after including a 1.5‐year lag.^14^

Our results are unlikely to be explained by indication bias, as analyses were adjusted for time‐varying CCBs indications. Moreover, our analyses were adjusted for the time‐varying number of consultations, which could be associated with better detection of hypertension/hypotension. Finally, different findings for non‐DiCCBs and DiCCBs argue against an indication bias because they share similar indications.

We distinguished CCBs according to their selectivity (DiCCBs/non‐DiCCBs) and ability to cross the blood–brain barrier (amlodipine/non‐amlodipine DiCCBs). One study with the same subgroups (2‐year lag) showed a statistically significant inverse association for non‐amlodipine DiCCBs (odds ratio [OR] = 0.73, 95% CI = 0.54–0.97), but no association for amlodipine (OR = 1.04, 95% CI% = 0.87–1.25) and non‐DiCCBs (OR = 1.14, 95% CI = 0.93–1.39).^12^ Several other studies distinguished DiCCBs from non‐DiCCBs without implementing a lag and reached conflicting findings for non‐DiCCBs.^9^, ^13^, ^15^

Two studies that investigated individual DiCCBs showed a positive association for amlodipine: one (no lag) reported reduced PD risk in amlodipine users compared with non‐users (OR = 0.70, 95% CI = 0.59–0.83),^13^ while the other reported a non‐significant inverse association compared with beta‐blockers (OR = 0.83, 95% CI = 0.67–1.03).^14^ No studies examined diltiazem or verapamil individually (non‐DiCCBs).

The positive association of amlodipine and non‐DiCCBs, especially diltiazem, with PD represents an original finding. Although there are differences in their ability to cross the brain–blood barrier,^7^ all CCBs, including amlodipine/diltiazem, can penetrate the brain, especially during chronic treatment.^4^, ^5^, ^6^ Flunarizine and cinnarizine, two non‐antihypertensive CCBs mainly used for vertigo, can induce parkinsonism or reveal underlying PD due to a piperazine nucleus that interferes with dopamine metabolism.^40^, ^41^ There are a few case reports of drug‐induced parkinsonism caused by amlodipine,^42^, ^43^ diltiazem,^44^, ^45^, ^46^, ^47^, ^48^ or verapamil,^45^, ^46^, ^49^, ^50^ that improved after drug discontinuation; these drugs are regularly included among possible causes of drug‐induced parkinsonism.^40^ One interpretation of our findings is that amlodipine and diltiazem aggravated motor symptoms in women with pre‐existing presynaptic dopaminergic denervation and accelerated the PD diagnosis. There was a difference in the relationship of amlodipine and non‐DiCCBs (including diltiazem) with PD. Whereas the association with amlodipine was restricted to analyses without a lag (ie, for relatively recent exposures), the association for non‐DiCCBs (including diltiazem) was stronger in analyses with a 5‐year lag. One possible explanation could be that longer exposure periods and higher cumulative doses may be required for non‐DiCCBs than amlodipine; however, this difference must be interpreted with caution, given the small number of exposed cases. We did not show any association for verapamil, but the small number of exposed cases makes it difficult to draw firm conclusions. These findings warrant replication in studies with longer lags in order to better examine the temporal relation between exposure to non‐DiCCBs and PD. If replicated, it remains to be demonstrated whether CCBs discontinuation in PD patients could, in some cases, improve symptoms.

The mechanisms involved in this association are unknown but limited experimental studies suggest that some CCBs, including verapamil and diltiazem (amlodipine not tested), can bind to D2‐dopamine receptors and reduce dopamine neurotransmission; in addition, there is some evidence that diltiazem (as well as flunarizine and cinnarizine) is toxic for dopamine cells.^51^ Additional studies are needed to determine potential mechanisms.

Strengths of our study lie in the large sample size and long follow‐up allowing reverse causation to be addressed by performing lagged analyses and trajectories analyses. We used a new‐user study design that allows reduction of the risk of survival bias for exposures associated with mortality.^32^, ^52^ Drugs were modeled as time‐varying exposures updated over the follow‐up to avoid immortal time bias and take into account changes in exposure.^53^ Validation of PD cases allowed vascular parkinsonism to be ruled out and minimized diagnostic misclassification.^20^ Finally, we adjusted for a wide range of characteristics to reduce the risk of residual confounding and indication bias.

Our study has limitations. First, it mostly includes health‐conscious women who are not representative of the general population. E3N participants are educated and motivated women who provide high‐quality information in questionnaires with high response rates.^18^ However, the selected nature of the cohort does not appear to have led to lower rates.^20^ Second, no conclusions can be drawn regarding men. Nevertheless, women are under‐represented in PD research and studies in this population are needed. Third, exploratory subgroup analyses must be interpreted with caution due to the small number of exposed PD patients. We were also unable to perform dose–response analyses for individual drugs and to examine characteristics (other than cumulative duration/dose) that would explain why some CCBs users develop PD; additional studies are needed to understand which factors drive susceptibility to CCBs. Fourth, we were unable to perform survival analyses with lags longer than 7 years that would be useful to better characterize the temporal relation between exposure to non‐DiCCBs/diltiazem and PD. However, trajectory analyses showing no differences in prescriptions of non‐DiCCBs between cases and controls 8–10 years before diagnosis suggest that distant exposure is less likely to be associated with PD compared with more recent exposure. Fifth, although analyses were adjusted for the main CCBs indications, the exact indication for each participant was not available. Sixth, the algorithm we used in the absence of medical records has imperfect specificity. We performed sensitivity analyses using a more specific version of the algorithm; in general, reducing diagnostic misclassification led to stronger associations.

In conclusion, our study found no evidence in favor of a neuroprotective role of CCBs for PD. Conversely, one possible explanation for our findings for amlodipine and non‐DiCCBs (notably diltiazem) is that these drugs may accelerate the development of PD in patients with pre‐existing presynaptic dopaminergic denervation.

## Author Roles

(1) Research Project: A. Design, B. Organization, C. Execution; (2) Statistical Analysis: A. Design, B. Execution, C. Review and Critique; (3) Manuscript Preparation: A. Writing of the First Draft, B. Editing of the Final Version.E.M.: 1A, 1C, 2B, 3A, 3B.

A.F.: 3B.

E.C.: 3B.

F.A.: 3B.

P.T.‐B.: 3B.

E.R.: 3B.

I.A.: 3B.

A.C.M.T.: 3B.

A.E.: 1A, 1C, 2B, 3A, 3B.

T.T.H.N.: 1A, 1C, 2B, 3A, 3B.

## Financial Disclosures

E.M. reports no disclosures. A.F. reports no disclosures. E.C. reports no disclosures. F.A. reports no disclosures. P.T.‐B. reports no disclosures. E.R. received honorarium for speaking from Orkyn, Aguettant, Elivie, Merz‐Pharma, Janssen, and Teva, and for participating on advisory boards for Merz‐Pharma, Elivie, and Teva. He received research support from Merz‐Pharma, Orkyn, Elivie, Everpharma, AMADYS, ADCY5.org, Fonds de dotation Patrick Brou de Laurière, Agence Nationale de la Recherche, Dystonia Medical Research Foundation, Hope For Annabel, Cure Alternating Hemiplegia of Childhood, Alternating Hemiplegia of Childhood Foundation, Alternating Hemiplegia of Childhood Association of Iceland, Association française de l'hémiplégie alternante, and Alternating Hemiplegia of Childhood Kids of the Netherlands. I.A. reports no disclosures. A.C.M.T. reports no disclosures. A.E. received honorarium for speaking from the Prada Foundation and has obtained research grants from Agence nationale de la recherche (ANR), The Michael J. Fox Foundation for Parkinson's Research, and France Parkinson. T.T.H.N. was supported by postdoctoral grants from The Michael J. Fox Foundation for Parkinson's Research, the French Ministry of Agriculture, and the France Parkinson Association.

## Supporting information


**Table S1.** List of calcium channel blockers.
**Table S2.** Source of Parkinson's disease patients.
**Table S3.** Baseline characteristics of the participants according to incident Parkinson's disease status at the end of follow‐up.
**Table S4.** Association of calcium channel blockers use with the incidence of Parkinson's disease: analyses with a 7‐year lag.
**Table S5.** Baseline participants' characteristics, overall, and by calcium channel blockers exposure at the end of follow‐up in analyses without an exposure lag.
**Table S6.** Association of calcium channel blockers use with the incidence of Parkinson's disease: analyses without a lag.
**Table S7.** Association of amlodipine, non‐amlodipine dihydropyridines (DiCCBs), and non‐dihydropyridines (non‐DiCCBs) use with the incidence of Parkinson's disease.
**Table S8.** Association of amlodipine, lercanidipine, verapamil, diltiazem, and other calcium channel blockers use with the incidence of Parkinson's disease: analyses without a lag.
**Table S9.** Association of time since first use and time since last use of calcium channel blockers with the incidence of Parkinson's disease: analyses without an exposure lag.
**Table S10.** Association of calcium channel blockers use with the incidence of Parkinson's disease including prevalent users: analyses.
**Table S11.** Association of calcium channel blockers use with the incidence of Parkinson's disease: analyses with a 5‐year exposure lag.
**Table S12.** Association of calcium channel blockers use with the incidence of Parkinson's disease: analyses with a more specific version of the algorithm (specificity=sensitivity=90%).
**Table S13.** Case–control studies on the association between use of calcium channel blockers and incidence of Parkinson's disease.
**Table S14.** Cohort studies on the association between use of calcium channel blockers and incidence of Parkinson's disease.
**Figure S1.** Study design for the analysis of the relation of calcium channel blockers use with the incidence of Parkinson's disease with a 5‐year lag.
**Figure S2.** Study design for the analysis of the relation of calcium channel blockers use with the incidence of Parkinson's disease without an exposure lag.
**Figure S3.** Directed acyclic graph showing the hypothesized causal structure of the variables included in the analyses.

## Data Availability

E3N data are available to bona fide researchers. Data are made available under managed access owing to governance constraints and the need to protect the privacy of participants. Data requests should be submitted online (https://www.e3n-generations.fr/) or sent to contact@e3n.fr.
