# Supplementary material for: Revisiting the Association Between Calcium Channel Blockers and Parkinson's Disease in the E3N Cohort
Source: Mov Disord. 2026 Apr 29;41(8):2071–85. doi: 10.1002/mds.70336 (PMC13518259; doi:10.1002/mds.70336)
Supplement: Supplementary file 1 [file MDS-41-2071-s001.pdf]

Supplementary methods

Revisiting the association between calcium channel blockers and Parkinson's disease in the E3N cohort

Covariates

Our analyses were adjusted for covariates known to be associated with Parkinson's disease. Several previous studies have shown that high caffeine consumption,[1] smoking,[1] obesity,[2] high physical activity,[3] and gout[4] are associated with a lower incidence of Parkinson's disease. Conversely, rural residence,[1] high number of children,[5] higher level of education,[6] early or late age at menarche,[5] artificial menopause,[5] and type 2 diabetes[7] are associated with a higher incidence of Parkinson's disease.

Type of residence (rural, urban), education level (<high school/up to 2 university y/>2 university y), parity (nulliparous/1/2/≥3 children), and age at menarche (<12/12/≥13y) were reported at baseline. Caffeine consumption (mg/day, in quartiles) was assessed at Q3 (1993). Time-varying covariates evaluated over the follow-up included: smoking (never/former/current smoker), BMI (<18.5/18.5-24.9/25.0-29.9/≥30.0 kg/m²), menopausal status (pre- or natural/artificial/unknown type), and constipation (yes/no). Physical activity (MET-h / week) was assessed 6 times during follow-up (Q1-1990, Q3-1993, Q5-1997, Q7-2002, Q8-2005 and Q11-2014). Since the questions differed from one questionnaire to the next, a latent process mixed model with random intercept and slope was used to create a latent variable (categorized into quartiles in the analyses) that captures the non-Gaussian longitudinal trajectory of physical activity.[3] Time-varying history of type 2 diabetes and gout was determined based on self-reports from questionnaires and/or drug claims databases (antidiabetics/anti-gout drugs).

In addition, our analyses were also adjusted for comorbidities that represent main indications of CCBs, to address the potential for indication bias. We used self-reports from questionnaires to define time-varying comorbidities associated with CCBs use (hypertension and cardiovascular disease including ischemic heart disease, stroke, angina). In addition, although unfrequently, CCBs can be used to treat cardiac arrhythmia, in particular atrial fibrillation (AF) when beta-blockers are not indicated or tolerated.[8] Constipation is a common prodromal symptom of PD.[1] Diltiazem and less frequently nifedipine can be used to treat anal fissures, which are most commonly caused by constipation; in this case, CCBs are generally prescribed as topical creams or ointments, and exceptionally as pills.

We used MGEN drug claims databases to assess time-varying use of lipophilic statins[9] (ATC-C10AA/C10BA/C10BX) and long/ultra-long-acting β2-agonists (ATC-R03)[10] as they were associated with PD risk in E3N.

Use of medical services should be considered when evaluating associations of exposures with PD to ensure that they are not attributable to bias. We used healthcare databases to compute the number of medical contacts during the preceding six months (updated each month over the follow-up) and included this variable as a time-varying covariate in the analyses.[11]

A directed acyclic graph (DAG) shows the hypothetical causal structure of the variables; all the variables included in the model represent common causes and therefore confounders of the exposure-outcome association (Figure S3).

Sensitivity analyses

- We performed analyses with a 7y-lag and without a lag for comparison to our main analyses. For analyses without a lag, we examined time since first (<2.5y, 2.5-4.9y, ≥5.0y) or last use (current use or cessation<2.5y, cessation between 2.5-4.9y, cessation≥5.0y). These cutoffs were chosen in order to include a similar number of exposed subjects in each group.
- We performed analyses that included prevalent CCBs users (5y-lag). We only considered ever use since starting date of use is unknown for prevalent users.
- We performed analyses adjusted for uncommon CCBs indications (cardiac arrhythmia, constipation). CCBs can be prescribed for cardiac arrhythmia, especially atrial fibrillation when other treatments are not indicated or tolerated. CCBs can also be prescribed to treat anal fissures, which are most commonly caused by constipation; they are generally prescribed as topical creams/ointments and exceptionally as pills.
- The algorithm used in the absence of medical records has imperfect specificity. We examined the impact of using another version of the algorithm with higher specificity (specificity=sensitivity=90%). We hypothesized that reducing the number of false positives would increase the strength of the associations.

Table S1 | List of calcium channel blockers.

Calcium channel blockers	Molecule	Anatomical Therapeutic Chemical code	5y-lag N exposed (N cases)	No lag N exposed (N cases)
Dihydropyridines	Amlodipine	C08CA01	5,139 (22)	10,338 (78)
	Felodipine	C08CA02	376 (1)	559 (5)
	Isradipine	C08CA03	195 (1)	279 (2)
	Nicardipine	C08CA04	1,831 (6)	4,225 (26)
	Nifedipine	C08CA05	469 (2)	647 (6)
	Nimodipine	C08CA06	9 (0)	13 (0)
	Nitrendipine	C08CA08	99 (0)	146 (1)
	Lacidipine	C08CA09	115 (0)	144 (0)
	Manidipine	C08CA11	761 (2)	1,080 (7)
	Lercanidipine	C08CA13	5,129 (28)	9,170 (56)
Non-dihydropyridines	Verapamil	C08DA01	1,843 (11)	2,707 (22)
	Diltiazem	C08DB01	1,080 (11)	1,572 (19)
	Bepridil	C08EA02	18 (0)	21 (0)

Table S2 | Source of Parkinson's disease (PD) patients.

Source of PD patients	No lag	5-year lag
**Medical records**	545 (65%)	406 (65%)
**Algorithm**	302 (35%)	221 (35%)
**Total**	847	627
**Medical records (without Possible PD)**	504 (62%)	382 (62%)
**Algorithm**	302 (38%)	221 (37%)
**Total**	806	603
**Medical records**	545	406
**Definite PD**	430 (79%)	326 (80%)
**Probable PD**	74 (14%)	56 (14%)
**Possible PD**	41 (7%)	24 (6%)

Table S3 | Baseline characteristics of the participants according to incident PD status at the end of follow-up.

Characteristics in 2004, N (%)	Parkinson's disease at the end of follow-up Yes (N=603)	No (N=82,002)
Mean (SD) age (years)	64.9 (5.9)	62.7 (6.3)
Residence		
Rural	73 (13.3)	11,087 (14.8)
Urban	475 (86.7)	63,750 (85.2)
Missing	*55 (9.1)*	*7165 (8.7)*
Education		
< High school	67 (11.6)	9,002 (11.5)
Up to 2 university years	303 (52.2)	40,066 (51.0)
> 2 university years	210 (36.2)	29,488 (37.5)
Missing	*23 (3.8)*	*3,446 (4.2)*
Parity		
Nulliparous	73 (12.2)	9,941 (12.2)
1 child	80 (13.4)	13,450 (16.5)
2 children	263 (44.0)	35,329 (43.4)
≥ 3 children	182 (30.4)	22,672 (27.9)
Missing	*5 (0.8)*	*610 (0.7)*
Age at menarche (years)		
< 12	133 (22.8)	16,770 (21.0)
12	124 (21.3)	20,341 (25.4)
≥ 13	326 (55.9)	42,932 (53.6)
Missing	*20 (3.3)*	*1,959 (2.4)*
Caffeine consumption (mg/day)		
< 94	125 (26.7)	15,614 (24.9)
94-173	112 (23.9)	15,526 (24.7)
174-270	128 (27.4)	15,703 (25.0)
≥ 271	103 (22.0)	15,972 (25.4)
Missing	*135 (22.4)*	*19,187 (23.4)*
Smoking status		
Non-smoker	367 (60.9)	43,421 (52.9)
Former smoker	192 (31.8)	29,350 (35.8)
Current smoker	44 (7.3)	9,231 (11.3)
Body mass index (kg/m²)		
< 18.5	18 (3.0)	2,592 (3.1)
18.5-24.9	409 (67.8)	54,942 (67.1)
25.0-29.9	146 (24.2)	19,078 (23.3)
≥ 30.0	30 (5.0)	5,306 (6.5)
Missing	*0 (0.0)*	*84 (0.1)*
Physical activity[a]		
Quartile1	153 (25.4)	20,467 (25.0)
Quartile2	151 (25.1)	20,469 (25.0)
Quartile3	160 (26.6)	20,460 (25.0)
Quartile4	138 (22.9)	20,482 (25.0)
Missing	*1 (0.0)*	*124 (0.1)*
Menopause		
No	2 (0.3)	1,312 (1.7)
Natural	497 (85.1)	66,909 (84.8)
Artificial	71 (12.2)	7,603 (9.6)
Unknown type	14 (2.4)	3,074 (3.9)
Missing	*19 (3.2)*	*3,104 (3.8)*
Number of consultations[b]		
< 2	92 (15.3)	12,940 (15.8)
2-3	176 (29.2)	22,918 (27.9)
4-6	192 (31.8)	25,482 (31.1)
≥ 7	143 (23.7)	20,662 (25.2)
Comorbidities		
Type 2 diabetes	18 (3.0)	2,266 (2.8)
Hypertension	165 (27.4)	21,527 (26.3)
Cardiovascular disease	16 (2.7)	1,683 (2.1)
Gout	11 (1.8)	1,169 (1.4)
Constipation	152 (25.2)	17,885 (21.2)
Cardiac arrhythmia	31 (6.6)	3,720 (5.9)
Missing	*134 (22.2)*	*19,168 (23.4)*
Comedication (at least one use)		
Lipophilic statins	65 (10.8)	8,992 (11.0)
Beta2 agonists (LABA and ultra-LABA)	28 (4.6)	3,100 (3.8)

SD, standard deviation; CCBs, calcium channels blocker; LABA, long-acting.

[a] Total physical activity defined based on a latent class mixed model and categorized into quartiles.

[b] Evaluated during the first 6 months of 2004.

Table S4 | Association of CCBs use with the incidence of Parkinson's disease: analyses with a 7-year lag.

CCBs	Cases N=495	IR	Model 1 HR (95% CI)[a]	p-value	Model 2 HR (95% CI)[b]	p-value	Model 3 HR (95% CI)[c]	p-value
CCBs								
Never users	460	0.83 (0.76-0.91)	1.00 (Reference)	0.88	1.00 (Reference)	0.91	1.00 (Reference)	0.98
Ever users	35	1.00 (0.72-1.39)	0.97 (0.69-1.38)		0.98 (0.69-1.39)		1.01 (0.70-1.44)	
**Mutually adjusted model A**								
**Amlodipine**								
Never users	483	0.84 (0.77-0.92)	1.00 (Reference)	0.79	1.00 (Reference)	0.77	1.00 (Reference)	0.73
Ever users	12	1.12 (0.63-1.96)	1.08 (0.60-1.95)		1.09 (0.61-1.96)		1.11 (0.62-2.00)	
**Non-amlodipine DiCCBs**								
Never users	476	0.84 (0.77-0.92)	1.00 (Reference)	0.57	1.00 (Reference)	0.58	1.00 (Reference)	0.61
Ever users	19	0.92 (0.59-1.45)	0.87 (0.54-1.39)		0.88 (0.55-1.40)		0.88 (0.55-1.42)	
**Non DiCCBs**								
Never users	485	0.84 (0.77-0.92)	1.00 (Reference)	0.61	1.00 (Reference)	0.60	1.00 (Reference)	0.48
Ever users	10	1.16 (0.62-2.15)	1.18 (0.63-2.22)		1.19 (0.63-2.23)		1.26 (0.67-2.37)	
**Mutually adjusted model B**								
**Amlodipine**								
Never users	483	0.84 (0.77-0.92)	1.00 (Reference)	0.82	1.00 (Reference)	0.80	1.00 (Reference)	0.75
Ever users	12	1.12 (0.63-1.96)	1.07 (0.60-1.92)		1.08 (0.60-1.94)		1.10 (0.61-1.99)	
**Lercanidipine**								
Never users	481	0.84 (0.77-0.92)	1.00 (Reference)	0.77	1.00 (Reference)	0.76	1.00 (Reference)	0.74
Ever users	14	1.12 (0.66-1.89)	1.09 (0.63-1.87)		1.09 (0.63-1.88)		1.10 (0.63-1.90)	
**Verapamil**								
Never users	491	0.85 (0.77-0.92)	1.00 (Reference)	0.41	1.00 (Reference)	0.41	1.00 (Reference)	0.47
Ever users	4	0.70 (0.26-1.86)	0.66 (0.24-1.78)		0.66 (0.24-1.78)		0.69 (0.26-1.88)	
**Diltiazem**								
Never users	488	0.84 (0.77-0.91)	1.00 (Reference)	0.03	1.00 (Reference)	0.03	1.00 (Reference)	0.02
Ever users	7	2.18 (1.04-4.57)	2.29 (1.08-4.86)		2.29 (1.08-4.87)		2.44 (1.14-5.21)	
**Others CCBs**								
Never users	488	0.85 (0.77-0.92)	1.00 (Reference)	0.31	1.00 (Reference)	0.32	1.00 (Reference)	0.33
Ever users	7	0.72 (0.35-1.52)	0.68 (0.32-1.44)		0.68 (0.32-1.45)		0.69 (0.32-1.47)	

CCBs, calcium channel blocker; IR, incidence rate (per 1,000 person-years); HR, hazard ratio; CI, confidence interval; DDD, defined daily dose.

[a] HRs, 95% CIs, and P-values were computed using Cox proportional hazards regression for time-varying variables with age as the timescale. Amlodipine, non-amlodpine DiCCBs (felodipine, isradipine, lacidipine, manidipine, nicardipine, nifedipine, nimodipine, and lercanidipine) and non-DiCCBs (bepridil, verapamil, and ditiazem) were included in the same model (mutually adjusted model A). Amlodipine, lercanidipine, verapamil, ditiazem and other calcium channel blockers (felodipine, isradipine, lacidipine, manidipine, nicardipine, nifedipine, nimodipine, bepridil) were included in the same model (mutually adjusted model B).

[b] Model 1 further adjusted for time-invariant variables: type of residence, education level, parity, age at first menarche, and caffeine consumption, and for time-varying variables: smoking status, body mass index, physical activity level, menopausal status, number of consultations in the previous 6 months, type 2 diabetes and gout.

[c] Model 2 further adjusted for time-varying cardiovascular diseases, hypertension, lipophilic statins and beta2 agonists (LABA and ultra-LABA).

Table S5 | Baseline participants' characteristics, overall and by CCBs exposure at the end of follow-up in analyses without an exposure lag.

Characteristics in 2004, N (%)	All women (N=84,909)	Calcium channel blocker exposure at the end of follow-up: Ever users (N=21,832)[a]	Calcium channel blocker exposure at the end of follow-up: Never users (N=63,077)
Mean (SD) age (years)	62.8 (6.4)	65.0 (6.6)	62.1 (6.1)
Residence			
Rural	11,444 (14.8)	2,813 (14.1)	8,631 (15.0)
Urban	66,042 (85.2)	17,119 (85.9)	48,923 (85.0)
Missing	*7,423 (8.7)*	*1,900 (8.7)*	*5,523 (8.8)*
Education			
< High school	9,452 (11.6)	2,840 (13.6)	6,612 (10.9)
Up to 2 university years	41,451 (51.0)	11,138 (53.3)	30,313 (50.1)
> 2 university years	30,449 (37.4)	6,904 (33.1)	23,545 (39.0)
Missing	*3,557 (4.2)*	*950 (4.4)*	*2,607 (4.1)*
Parity			
Nulliparous	10,394 (12.4)	2,582 (11.9)	7,812 (12.5)
1 child	13,915 (16.5)	3,479 (16.1)	10,436 (16.7)
2 children	36,433 (43.2)	8,911 (41.2)	27,522 (43.9)
≥ 3 children	23,521 (27.9)	6,675 (30.8)	16,846 (26.9)
Missing	*646 (0.8)*	*185 (0.8)*	*461 (0.7)*
Age at menarche (years)			
< 12	17,387 (21.0)	4,579 (21.5)	12,808 (20.8)
12	21,004 (25.3)	5,399 (25.4)	15,605 (25.3)
≥ 13	44,483 (53.7)	11,319 (53.1)	33,164 (53.9)
Missing	*2,035 (2.4)*	*535 (2.5)*	*1,500 (2.4)*
Caffeine consumption (mg/day)			
< 94	16,166 (24.9)	4,111 (25.1)	12,055 (24.9)
94-173	16,066 (24.7)	4,124 (25.1)	11,942 (24.6)
174-270	16,214 (25.0)	4,117 (25.1)	12,097 (24.9)
≥ 271	16,467 (25.4)	4,059 (24.7)	12,408 (25.6)
Missing	*19,996 (23.5)*	*5,421 (24.5)*	*14,575 (23.8)*
Smoking status			
Non-smoker	45,023 (53.0)	12,072 (55.3)	32,951 (52.2)
Former smoker	30,298 (35.7)	7,468 (34.2)	22,830 (36.2)
Current smoker	9,588 (11.3)	2,292 (10.5)	7,296 (11.6)
Body mass index (kg/m²)			
< 18.5	2,725 (3.2)	578 (2.7)	2,147 (3.4)
18.5-24.9	56,765 (66.9)	12,952 (59.4)	43,813 (69.5)
25.0-29.9	19,793 (23.4)	6,177 (28.3)	13,616 (21.6)
≥ 30.0	5,539 (6.5)	2,102 (9.6)	3,437 (5.5)
Missing	*87 (0.1)*	*23 (0.1)*	*64 (0.1)*
Physical activity[b]			
Quartile1	21,194 (25.0)	5,772 (26.5)	15,422 (24.5)
Quartile2	21,194 (25.0)	5,377 (24.7)	15,817 (25.1)
Quartile3	21,194 (25.0)	5,215 (23.9)	15,979 (25.4)
Quartile4	21,194 (25.0)	5,429 (24.9)	15,765 (25.0)
Missing	*133 (0.1)*	*39 (0.2)*	*94 (0.1)*
Menopause			
No	1,319 (1.6)	230 (1.1)	1,089 (1.8)
Natural	69,104 (84.6)	17,623 (83.5)	51,481 (85.0)
Artificial	8,044 (9.9)	2,513 (11.9)	5,531 (9.1)
Unknown type	3,204 (3.9)	750 (3.5)	2,454 (4.1)
Missing	*3,238 (3.8)*	*716 (3.3)*	*2,522 (4.0)*
Number of consultations[c]			
< 2	13,336 (15.7)	2,514 (11.5)	10,822 (17.1)
2-3	23,560 (27.7)	5,522 (25.3)	18,038 (28.6)
4-6	26,309 (31.0)	7,208 (33.0)	19,101 (30.3)
≥ 7	21,704 (25.6)	6,588 (30.2)	15,116 (24.0)
Comorbidities			
Type 2 diabetes	2,415 (2.8)	1,098 (5.0)	1,317 (2.1)
Hypertension	22,405 (26.4)	10,091 (46.2)	12,314 (19.5)
Cardiovascular disease	1,770 (2.1)	733 (3.4)	1,037 (1.6)
Gout	1,248 (1.5)	514 (2.4)	734 (1.2)
Comedication (at least one use)			
Lipophilic statins	9,352 (11.0)	3,132 (14.3)	6,220 (9.9)
Beta2 agonists (LABA and ultra-LABA)	3,281 (3.9)	1,114 (5.1)	2,167 (3.4)

SD, standard deviation; CCBs, calcium channels blocker; LABA, long-acting.

[a] Women exposed to CCBs at least once during follow-up

[b] Total physical activity defined based on a latent class mixed model and categorized into quartiles.

[c] Evaluated during the first 6 months of 2004.

Table S6 | Association of CCBs use with the incidence of Parkinson's disease: analyses without a lag.

CCBs	Cases N=806	IR	Model 1 HR (95% CI)[a]	p-value	Model 2 HR (95% CI)[b]	p-value	Model 3 HR (95% CI)[c]	p-value
Never users	651	0.64 (0.59-0.69)	1.00 (Reference)	0.08	1.00 (Reference)	0.19	1.00 (Reference)	0.10
Ever users	155	1.07 (0.91-1.25)	1.17 (0.98-1.40)		1.13 (0.94-1.36)		1.18 (0.97-1.43)	
**Cumulative dose (DDDs)**								
Never	651	0.64 (0.59-0.69)	1.00 (Reference)	0.12[d]	1.00 (Reference)	0.12[d]	1.00 (Reference)	0.07[d]
< 90	44	0.95 (0.71-1.28)	1.11 (0.81-1.51)		1.05 (0.77-1.43)		1.08 (0.79-1.48)	
90-761	53	1.05 (0.81-1.38)	1.16 (0.87-1.54)		1.10 (0.83-1.46)		1.14 (0.86-1.53)	
≥ 762	58	1.20 (0.93-1.55)	1.24 (0.95-1.63)		1.24 (0.94-1.64)		1.31 (0.99-1.75)	
**Cumulative duration (days)**								
Never	651	0.64 (0.59-0.69)	1.00 (Reference)	0.10[d]	1.00 (Reference)	0.11[d]	1.00 (Reference)	0.06[d]
< 90	47	1.00 (0.75-1.34)	1.17 (0.87-1.57)		1.11 (0.82-1.49)		1.14 (0.84-1.54)	
90-727	49	0.99 (0.75-1.31)	1.09 (0.81-1.46)		1.03 (0.77-1.38)		1.07 (0.79-1.45)	
≥ 728	59	1.22 (0.95-1.58)	1.26 (0.96-1.66)		1.26 (0.96-1.66)		1.33 (1.00-1.77)	
**Average daily dose (DDDs)**								
Never	651	0.64 (0.59-0.69)	1.00 (Reference)	0.14[d]	1.00 (Reference)	0.27[d]	1.00 (Reference)	0.15[d]
< 1	34	1.44 (1.03-2.02)	1.60 (1.13-2.27)		1.48 (1.04-2.09)		1.54 (1.08-2.19)	
= 1	79	1.00 (0.80-1.25)	1.11 (0.88-1.41)		1.08 (0.85-1.37)		1.12 (0.88-1.43)	
> 1	42	1.00 (0.74-1.35)	1.05 (0.76-1.44)		1.02 (0.74-1.40)		1.07 (0.77-1.48)	

CCBs, calcium channel blocker; IR, incidence rate (per 1,000 person-years); HR, hazard ratio; CI, confidence interval; DDD, defined daily dose.

[a] HRs, 95% CIs, and P-values were computed using Cox proportional hazards regression for time-varying variables with age as the timescale.

[b] Model 1 further adjusted for time-invariant variables: type of residence, education level, parity, age at first menarche, and caffeine consumption, and for time-varying variables: smoking status, body mass index, physical activity level, menopausal status, number of consultations in the previous 6 months, type 2 diabetes and gout.

[c] Model 2 further adjusted for time-varying cardiovascular diseases, hypertension, lipophilic statins and beta2 agonists (LABA and ultra-LABA).

[d] P values for linear trend.

Table S7 | Association of amlodipine, non-amlodipine DiCCBs and non-DiCCBs use with the incidence of Parkinson's disease: analyses without a lag.

CCBs	Cases N=806	IR	Model 1 HR (95% CI)[a]	p-value	Model 2 HR (95% CI)[b]	p-value	Model 3 HR (95% CI)[c]	p-value
**Amlodipine**								
Never users	728	0.66 (0.61-0.71)	1.00 (Reference)	0.01	1.00 (Reference)	0.01	1.00 (Reference)	<0.01
Ever users	78	1.35 (1.08-1.68)	1.39 (1.09-1.78)		1.39 (1.09-1.78)		1.44 (1.12-1.85)	
Cumulative dose (DDDs)								
Never	728	0.66 (0.61-0.71)	1.00 (Reference)	0.02[d]	1.00 (Reference)	0.01[d]	1.00 (Reference)	0.01[d]
≤ 199	37	1.28 (0.93-1.77)	1.33 (0.95-1.88)		1.30 (0.92-1.83)		1.34 (0.95-1.89)	
> 199	41	1.42 (1.05-1.93)	1.45 (1.05-2.00)		1.48 (1.07-2.05)		1.54 (1.11-2.15)	
Cumulative duration (days)								
Never	728	0.66 (0.61-0.71)	1.00 (Reference)	0.01[d]	1.00 (Reference)	0.01[d]	1.00 (Reference)	<0.01[d]
≤ 181	36	1.25 (0.90-1.73)	1.30 (0.92-1.83)		1.27 (0.90-1.79)		1.30 (0.92-1.85)	
> 181	42	1.45 (1.07-1.97)	1.49 (1.08-2.04)		1.52 (1.10-2.09)		1.58 (1.14-2.19)	
Average daily dose (DDDs)								
Never	728	0.66 (0.61-0.71)	1.00 (Reference)	0.01[d]	1.00 (Reference)	0.01[d]	1.00 (Reference)	0.01[d]
≤ 1	63	1.37 (1.07-1.75)	1.42 (1.09-1.86)		1.41 (1.08-1.85)		1.46 (1.11-1.92)	
> 1	15	1.28 (0.77-2.12)	1.31 (0.78-2.20)		1.33 (0.79-2.23)		1.38 (0.82-2.32)	
**Non-amlodipine DiCCBs**								
Never users	714	0.66 (0.62-0.71)	1.00 (Reference)	0.94	1.00 (Reference)	0.85	1.00 (Reference)	0.93
Ever users	92	1.05 (0.85-1.28)	1.01 (0.80-1.27)		0.98 (0.78-1.23)		1.01 (0.80-1.28)	
Cumulative dose (DDDs)								
Never	714	0.66 (0.62-0.71)	1.00 (Reference)	0.62[d]	1.00 (Reference)	0.58[d]	1.00 (Reference)	0.72[d]
≤ 214	50	1.14 (0.86-1.50)	1.12 (0.84-1.51)		1.07 (0.79-1.44)		1.10 (0.81-1.48)	
> 214	42	0.96 (0.71-1.29)	0.90 (0.65-1.24)		0.89 (0.65-1.23)		0.93 (0.67-1.29)	
Cumulative duration (days)								
Never	714	0.66 (0.62-0.71)	1.00 (Reference)	0.51[d]	1.00 (Reference)	0.47[d]	1.00 (Reference)	0.61[d]
≤ 199	51	1.16 (0.88-1.53)	1.15 (0.85-1.54)		1.09 (0.81-1.46)		1.12 (0.83-1.51)	
> 199	41	0.93 (0.69-1.27)	0.88 (0.64-1.22)		0.87 (0.63-1.21)		0.91 (0.65-1.26)	
Average daily dose (DDDs)								
Never	714	0.66 (0.62-0.71)	1.00 (Reference)	0.96[d]	1.00 (Reference)	0.83[d]	1.00 (Reference)	0.95[d]
≤ 1	64	1.09 (0.85-1.39)	1.07 (0.82-1.40)		1.03 (0.79-1.35)		1.07 (0.81-1.40)	
> 1	28	0.96 (0.66-1.39)	0.90 (0.61-1.32)		0.88 (0.60-1.30)		0.91 (0.62-1.35)	
**Non-DiCCBs**								
Never users	768	0.68 (0.63-0.73)	1.00 (Reference)	0.09	1.00 (Reference)	0.16	1.00 (Reference)	0.10
Ever users	38	1.24 (0.91-1.71)	1.34 (0.96-1.86)		1.27 (0.91-1.76)		1.32 (0.95-1.85)	
Cumulative dose (DDDs)								
Never	768	0.68 (0.63-0.73)	1.00 (Reference)	0.19[d]	1.00 (Reference)	0.29[d]	1.00 (Reference)	0.21[d]
≤ 117	19	1.24 (0.79-1.95)	1.35 (0.85-2.14)		1.28 (0.81-2.03)		1.32 (0.84-2.10)	
> 117	19	1.25 (0.79-1.95)	1.31 (0.83-2.07)		1.25 (0.79-1.98)		1.32 (0.83-2.09)	
Cumulative duration (days)								
Never	768	0.68 (0.63-0.73)	1.00 (Reference)	0.12[d]	1.00 (Reference)	0.19[d]	1.00 (Reference)	0.13[d]
≤ 130	18	1.18 (0.74-1.87)	1.28 (0.80-2.05)		1.21 (0.76-1.94)		1.25 (0.78-2.01)	
> 130	20	1.31 (0.85-2.03)	1.38 (0.88-2.15)		1.32 (0.84-2.06)		1.39 (0.89-2.18)	
Average daily dose (DDDs)								
Never	768	0.68 (0.63-0.73)	1.00 (Reference)	0.11[d]	1.00 (Reference)	0.20[d]	1.00 (Reference)	0.13[d]
< 1	20	1.61 (1.04-2.50)	1.73 (1.11-2.71)		1.60 (1.02-2.50)		1.67 (1.06-2.61)	
≥ 1	18	0.99 (0.63-1.58)	1.06 (0.66-1.70)		1.03 (0.64-1.65)		1.07 (0.67-1.72)	

CCBs, calcium channel blocker; DiCCBs, dihydropyridine calcium channel blocker; IR, incidence rate (per 1,000 person-years); HR, hazard ratio; CI, confidence interval; DDD, defined daily dose.

[a] HRs, 95% CIs, and P-values were computed using Cox proportional hazards regression for time-varying variables with age as the timescale. Amlodipine, non-amlodpine DiCCBs (felodipine, isradipine, lacidipine, manidipine, nicardipine, nifedipine, nimodipine, and lercanidipine) and non-DiCCBs (bepridil, verapamil, and ditiazem) were included in the same mutually adjusted model.

[b] Model 1 further adjusted for time-invariant variables: type of residence, education level, parity, age at first menarche, and caffeine consumption, and for time-varying variables: smoking status, body mass index, physical activity level, menopausal status, number of consultations in the previous 6 months, type 2 diabetes and gout.

[c] Model 2 further adjusted for time-varying cardiovascular diseases, hypertension, lipophilic statins and beta2 agonists (LABA and ultra-LABA).

[d] P values for linear trend.

Table S8 | Association of amlodipine, lercanidipine, verapamil, diltiazem, and other CCBs use with the incidence of Parkinson's disease: analyses without a lag.

CCBs	Cases N=806	IR	Model 1 HR (95% CI)[a]	p-value	Model 2 HR (95% CI)[b]	p-value	Model 3 HR (95% CI)[c]	p-value
**Amlodipine**								
Never users	728	0.66 (0.61-0.71)	1.00 (Reference)	0.01	1.00 (Reference)	0.01	1.00 (Reference)	<0.01
Ever users	78	1.35 (1.08-1.68)	1.41 (1.10-1.80)		1.41 (1.10-1.80)		1.45 (1.13-1.87)	
**Lercanidipine**								
Never users	750	0.68 (0.63-0.73)	1.00 (Reference)	0.53	1.00 (Reference)	0.46	1.00 (Reference)	0.59
Ever users	56	0.98 (0.76-1.28)	0.91 (0.69-1.21)		0.90 (0.68-1.19)		0.92 (0.69-1.23)	
**Verapamil**								
Never users	784	0.68 (0.64-0.73)	1.00 (Reference)	0.71	1.00 (Reference)	0.84	1.00 (Reference)	0.72
Ever users	22	1.07 (0.71-1.63)	1.09 (0.70-1.67)		1.04 (0.68-1.61)		1.08 (0.70-1.67)	
**Diltiazem**								
Never users	787	0.68 (0.64-0.73)	1.00 (Reference)	0.03	1.00 (Reference)	0.06	1.00 (Reference)	0.04
Ever users	19	1.62 (1.03-2.53)	1.68 (1.06-2.67)		1.57 (0.99-2.49)		1.63 (1.02-2.60)	
**Others CCBs[d]**								
Never users	761	0.68 (0.63-0.73)	1.00 (Reference)	0.69	1.00 (Reference)	0.89	1.00 (Reference)	0.78
Ever users	45	1.10 (0.82-1.47)	1.07 (0.78-1.46)		1.02 (0.75-1.40)		1.05 (0.76-1.43)	

CCBs, calcium channel blocker; IR, incidence rate (per 1,000 person-years); HR, hazard ratio; CI, confidence interval; DDD, defined daily dose.

[a] HRs, 95% CIs, and P-values were computed using Cox proportional hazards regression for time-varying variables with age as the timescale. Amlodipine, lercanidipine, verapamil, ditiazem and other calcium channel blockers (felodipine, isradipine, lacidipine, manidipine, nicardipine, nifedipine, nimodipine, bepridil) were included in the same mutually adjusted model.

[b] Model 1 further adjusted for time-invariant variables: type of residence, education level, parity, age at first menarche, and caffeine consumption, and for time-varying variables: smoking status, body mass index, physical activity level, menopausal status, number of consultations in the previous 6 months, type 2 diabetes and gout.

[c] Model 2 further adjusted for time-varying cardiovascular diseases, hypertension, lipophilic statins and beta2 agonists (LABA and ultra-LABA).

[d] Felodipine, isradipine, lacidipine, manidipine, nicardipine, nifedipine, nimodipine, bepridil.

Table S9| Association of time since first use and time since last use of calcium channel blockers with the incidence of Parkinson's disease: analyses without an exposure lag.

CCBs	Cases N=806	IR	Model 1 HR (95% CI)[a]	p-value	Model 2 HR (95% CI)[b]	p-value	Model 3 HR (95% CI)[c]	p-value
**CCBs**								
Time since first use (years)								
Never	651	0.64 (0.59-0.69)	1.00 (Reference)	0.16[d]	1.00 (Reference)	0.17[d]	1.00 (Reference)	0.09[d]
< 2.5y	44	0.93 (0.69-1.25)	1.11 (0.81-1.50)		1.01 (0.74-1.38)		1.04 (0.76-1.42)	
2.5-4.9y	45	1.18 (0.88-1.57)	1.31 (0.97-1.78)		1.25 (0.92-1.70)		1.31 (0.96-1.78)	
≥ 5.0y	66	1.12 (0.88-1.42)	1.14 (0.88-1.47)		1.15 (0.88-1.49)		1.21 (0.92-1.59)	
Time since last use (years)								
Never	651	0.64 (0.59-0.69)	1.00 (Reference)	0.82[e]	1.00 (Reference)	0.58[e]	1.00 (Reference)	0.59[e]
Current users or < 2.5y	114	1.07 (0.89-1.28)	1.17 (0.96-1.44)		1.12 (0.91-1.37)		1.16 (0.94-1.44)	
2.5-4.9y	18	0.95 (0.60-1.51)	1.05 (0.66-1.68)		1.02 (0.64-1.64)		1.06 (0.66-1.71)	
≥ 5.0y	23	1.22 (0.81-1.84)	1.29 (0.85-1.96)		1.33 (0.87-2.02)		1.38 (0.90-2.11)	
**Mutually adjusted model**								
**Amlodipine**								
Time since first use (years)								
Never	728	0.66 (0.61-0.71)	1.00 (Reference)	0.13[d]	1.00 (Reference)	0.10[d]	1.00 (Reference)	0.07[d]
< 2.5y	30	1.38 (0.97-1.97)	1.50 (1.04-2.18)		1.43 (0.99-2.07)		1.47 (1.01-2.14)	
2.5-4.9y	25	1.54 (1.04-2.28)	1.61 (1.07-2.41)		1.60 (1.07-2.41)		1.66 (1.10-2.50)	
≥ 5.0y	23	1.16 (0.77-1.74)	1.13 (0.73-1.73)		1.18 (0.77-1.82)		1.23 (0.79-1.90)	
Time since last use (years)								
Never	728	0.66 (0.61-0.71)	1.00 (Reference)	0.44[e]	1.00 (Reference)	0.58[e]	1.00 (Reference)	0.58[e]
Current users or < 2.5y	56	1.41 (1.08-1.83)	1.48 (1.12-1.96)		1.45 (1.10-1.92)		1.50 (1.13-2.00)	
2.5-4.9y	11	1.20 (0.67-2.18)	1.23 (0.67-2.26)		1.24 (0.68-2.27)		1.28 (0.70-2.35)	
≥ 5.0y	11	1.24 (0.69-2.24)	1.18 (0.65-2.17)		1.25 (0.68-2.29)		1.29 (0.70-2.38)	
**Non-amlodipine DiCCBs**								
Time since first use (years)								
Never	714	0.66 (0.62-0.71)	1.00 (Reference)	0.79[d]	1.00 (Reference)	0.75[d]	1.00 (Reference)	0.92[d]
< 2.5y	33	1.12 (0.79-1.57)	1.19 (0.83-1.69)		1.10 (0.77-1.56)		1.13 (0.79-1.61)	
2.5-4.9y	22	0.94 (0.62-1.43)	0.91 (0.59-1.40)		0.87 (0.57-1.34)		0.90 (0.58-1.39)	
≥ 5.0y	37	1.06 (0.77-1.46)	0.95 (0.67-1.34)		0.96 (0.68-1.35)		0.99 (0.70-1.41)	
Time since last use (years)								
Never	714	0.66 (0.62-0.71)	1.00 (Reference)	0.68[e]	1.00 (Reference)	0.48[e]	1.00 (Reference)	0.47[e]
Current users or < 2.5y	62	1.03 (0.80-1.32)	1.03 (0.78-1.34)		0.98 (0.75-1.28)		1.01 (0.77-1.33)	
2.5-4.9y	10	0.73 (0.40-1.37)	0.69 (0.37-1.29)		0.67 (0.36-1.26)		0.69 (0.37-1.30)	
≥ 5.0y	20	1.40 (0.91-2.18)	1.26 (0.80-1.99)		1.30 (0.83-2.06)		1.35 (0.85-2.14)	
**Non-DiCCBs**								
Time since first use (years)								
Never	768	0.68 (0.63-0.73)	1.00 (Reference)	0.05[d]	1.00 (Reference)	0.07[d]	1.00 (Reference)	0.05[d]
< 2.5y	7	0.78 (0.37-1.64)	0.94 (0.45-1.99)		0.85 (0.40-1.79)		0.88 (0.42-1.85)	
2.5-4.9y	11	1.43 (0.79-2.57)	1.58 (0.87-2.88)		1.47 (0.81-2.68)		1.54 (0.84-2.80)	
≥ 5.0y	20	1.44 (0.93-2.23)	1.45 (0.92-2.27)		1.42 (0.90-2.23)		1.49 (0.95-2.34)	
Time since last use (years)								
Never	768	0.68 (0.63-0.73)	1.00 (Reference)	0.95[e]	1.00 (Reference)	0.76[e]	1.00 (Reference)	0.78[e]
Current users or < 2.5y	20	1.09 (0.71-1.69)	1.25 (0.80-1.95)		1.15 (0.73-1.79)		1.20 (0.77-1.88)	
2.5-4.9y	9	1.79 (0.93-3.44)	1.93 (1.00-3.74)		1.82 (0.94-3.53)		1.89 (0.97-3.66)	
≥ 5.0y	9	1.25 (0.65-2.40)	1.18 (0.61-2.29)		1.19 (0.61-2.31)		1.24 (0.64-2.40)	

CCBs, calcium channel blocker; DiCCBs, dihydropyridine calcium channel blocker; IR, incidence rate (per 1,000 person-years); HR, hazard ratio; CI, confidence interval; DDD, defined daily dose.

[a] HRs, 95% CIs, and P-values were computed using Cox proportional hazards regression for time-varying variables with age as the timescale. Amlodipine, non-amlodpine DiCCBs (felodipine, isradipine, lacidipine, manidipine, nicardipine, nifedipine, nimodipine, and lercanidipine), and non-DiCCBs (bepridil, verapamil, and ditiazem) were included in the same model (mutually adjusted model).

[b] Model 1 further adjusted for time-invariant variables (type of residence, education level, parity, age at first menarche, and caffeine consumption), and for time-varying variables (smoking status, body mass index, physical activity level, menopausal status, number of consultations in the previous 6 months, type 2 diabetes and gout).

[c] Model 2 further adjusted for time-varying cardiovascular diseases, hypertension, lipophilic statins, and beta2 agonists (LABA and ultra-LABA).

[d] P-value for linear trend.

[e] P-value for linear trend among exposed participants.

Table S10 | Association of CCBs use with the incidence of Parkinson's disease including prevalent users: analyses with a 5-year exposure lag.

CCBs	Cases N=675	IR	Model 1 HR (95% CI)[a]	p-value	Model 2 HR (95% CI)[b]	p-value	Model 3 HR (95% CI)[c]	p-value
**CCBs**								
Never users	568	0.78 (0.71-0.84)	1.00 (Reference)	0.62	1.00 (Reference)	0.58	1.00 (Reference)	0.54
Ever users	107	1.04 (0.86-1.26)	1.05 (0.85-1.30)		1.06 (0.86-1.32)		1.07 (0.86-1.35)	
**Mutually adjusted model A**								
**Amlodipine**								
Never users	643	0.80 (0.74-0.86)	1.00 (Reference)	0.81	1.00 (Reference)	0.87	1.00 (Reference)	0.95
Ever users	32	1.00 (0.71-1.42)	0.96 (0.67-1.38)		0.97 (0.67-1.40)		0.99 (0.68-1.43)	
**Non-amlodipine DiCCBs**								
Never users	617	0.79 (0.73-0.86)	1.00 (Reference)	0.90	1.00 (Reference)	0.94	1.00 (Reference)	0.90
Ever users	58	1.01 (0.78-1.30)	0.98 (0.74-1.30)		0.99 (0.75-1.31)		0.98 (0.74-1.31)	
**Non-DiCCBs**								
Never users	637	0.79 (0.73-0.86)	1.00 (Reference)	0.13	1.00 (Reference)	0.13	1.00 (Reference)	0.08
Ever users	38	1.23 (0.90-1.69)	1.29 (0.92-1.79)		1.29 (0.93-1.80)		1.35 (0.96-1.89)	
**Mutually adjusted model B**								
**Amlodipine**								
Never users	643	0.80 (0.74-0.86)	1.00 (Reference)	0.73	1.00 (Reference)	0.79	1.00 (Reference)	0.88
Ever users	32	1.00 (0.71-1.42)	0.94 (0.65-1.35)		0.95 (0.66-1.37)		0.97 (0.67-1.41)	
**Lercanidipine**								
Never users	637	0.79 (0.73-0.86)	1.00 (Reference)	0.25	1.00 (Reference)	0.24	1.00 (Reference)	0.26
Ever users	38	1.22 (0.89-1.68)	1.22 (0.87-1.71)		1.23 (0.87-1.72)		1.22 (0.87-1.72)	
**Verapamil**								
Never users	652	0.80 (0.74-0.86)	1.00 (Reference)	0.43	1.00 (Reference)	0.42	1.00 (Reference)	0.35
Ever users	23	1.17 (0.78-1.76)	1.18 (0.78-1.80)		1.19 (0.78-1.81)		1.22 (0.80-1.87)	
**Diltiazem**								
Never users	655	0.80 (0.74-0.86)	1.00 (Reference)	0.03	1.00 (Reference)	0.03	1.00 (Reference)	0.02
Ever users	20	1.63 (1.05-2.53)	1.65 (1.05-2.59)		1.65 (1.05-2.59)		1.75 (1.11-2.77)	
**Other CCBs[d]**								
Never users	648	0.81 (0.75-0.87)	1.00 (Reference)	0.23	1.00 (Reference)	0.24	1.00 (Reference)	0.24
Ever users	27	0.84 (0.58-1.22)	0.79 (0.53-1.16)		0.79 (0.53-1.17)		0.79 (0.53-1.17)	

CCBs, calcium channel blocker; DiCCBs, dihydropyridine calcium channel blocker; IR, incidence rate (per 1,000 person-years); HR, hazard ratio; CI, confidence interval.

[a] HRs, 95% CIs, and P-values were computed using Cox proportional hazards regression for time-varying variables with age as the timescale. Amlodipine, non-amlodpine DiCCBs (felodipine, isradipine, lacidipine, manidipine, nicardipine, nifedipine, nimodipine, and lercanidipine) and non-DiCCBs (bepridil, verapamil, and ditiazem) were included in the same model (mutually adjusted model A). Amlodipine, lercanidipine, verapamil, ditiazem and other calcium channel blockers (felodipine, isradipine, lacidipine, manidipine, nicardipine, nifedipine, nimodipine, bepridil) were included in the same model (mutually adjusted model B).

[b] Model 1 further adjusted for time-invariant variables: type of residence, education level, parity, age at first menarche, and caffeine consumption, and for time-varying variables: smoking status, body mass index, physical activity level, menopausal status, number of consultations in the previous 6 months, type 2 diabetes and gout.

[c] Model 2 further adjusted for time-varying cardiovascular diseases, hypertension, lipophilic statins and beta2 agonists (LABA and ultra-LABA).

[d] Felodipine, isradipine, lacidipine, manidipine, nicardipine, nifedipine, nimodipine, bepridil.

Table S11 | Association of CCBs use with the incidence of Parkinson's disease: analyses with a 5-year exposure lag: adjustment for constipation and cardiac arrhythmia.

Exposure	Adjusted for constipation HR (95% CI)[a]	p-value	Adjusted for cardiac arrhythmia HR (95% CI)[b]	p-value	Adjusted for constipation and cardiac arrhythmia HR (95% CI)[c]	p-value
CCB overall						
Ever vs. never users	1.14 (0.87-1.50)	0.34	1.13 (0.86-1.49)	0.37	1.14 (0.86-1.49)	0.37
Cumulative dose (DDDs)						
Never	1.00 (Reference)	0.44[d]	1.00 (Reference)	0.47[d]	1.00 (Reference)	0.46[d]
< 64	1.05 (0.66-1.67)		1.05 (0.66-1.66)		1.05 (0.66-1.66)	
64-505	1.20 (0.78-1.85)		1.19 (0.78-1.83)		1.20 (0.78-1.83)	
≥ 506	1.17 (0.76-1.80)		1.16 (0.75-1.79)		1.16 (0.75-1.79)	
Cumulative duration (days)						
Never	1.00 (Reference)	0.25[d]	1.00 (Reference)	0.27[d]	1.00 (Reference)	0.27[d]
< 60	1.05 (0.65-1.69)		1.04 (0.65-1.68)		1.04 (0.65-1.68)	
60-486	1.10 (0.71-1.71)		1.09 (0.71-1.69)		1.09 (0.71-1.69)	
≥ 487	1.27 (0.84-1.93)		1.26 (0.83-1.91)		1.26 (0.83-1.92)	
Average daily dose (DDDs)						
Never	1.00 (Reference)	0.47[d]	1.00 (Reference)	0.50[d]	1.00 (Reference)	0.50[d]
< 1	1.54 (0.90-2.63)		1.53 (0.89-2.61)		1.52 (0.89-2.61)	
= 1	1.12 (0.79-1.58)		1.12 (0.79-1.57)		1.12 (0.79-1.58)	
> 1	0.94 (0.55-1.61)		0.93 (0.54-1.60)		0.93 (0.54-1.60)	
Amlodipine, non-amlodipine DiCCBs, and Non-DiCCBs[e]						
**Amlodipine**						
Ever vs. never users	1.08 (0.69-1.68)	0.74	1.07 (0.69-1.67)	0.75	1.07 (0.69-1.67)	0.75
Cumulative dose (DDDs)						
Never	1.00 (Reference)	0.27[d]	1.00 (Reference)	0.27[d]	1.00 (Reference)	0.27[d]
≤ 146	0.79 (0.39-1.61)		0.79 (0.39-1.61)		0.79 (0.39-1.60)	
> 146	1.36 (0.79-2.35)		1.36 (0.79-2.34)		1.36 (0.79-2.35)	
Cumulative duration (days)						
Never	1.00 (Reference)	0.25[d]	1.00 (Reference)	0.26[d]	1.00 (Reference)	0.26[d]
≤ 134	0.79 (0.39-1.61)		0.79 (0.39-1.61)		0.79 (0.39-1.61)	
> 134	1.36 (0.79-2.35)		1.36 (0.79-2.34)		1.36 (0.79-2.34)	
Average daily dose (DDDs)						
Never	1.00 (Reference)	0.96[d]	1.00 (Reference)	0.95[d]	1.00 (Reference)	0.96[d]
≤ 1	1.27 (0.81-2.00)		1.27 (0.81-2.00)		1.27 (0.81-2.00)	
> 1	0.25 (0.04-1.81)		0.25 (0.04-1.79)		0.25 (0.04-1.80)	
**Non-amlodipine DiCCBs**						
Ever vs. never users	1.02 (0.72-1.45)	0.91	1.01 (0.71-1.44)	0.94	1.01 (0.71-1.44)	0.94
Cumulative dose (DDDs)						
Never	1.00 (Reference)	0.94[d]	1.00 (Reference)	0.96[d]	1.00 (Reference)	0.96[d]
≤ 162	1.02 (0.63-1.65)		1.02 (0.63-1.64)		1.02 (0.63-1.64)	
> 162	1.03 (0.64-1.66)		1.03 (0.64-1.65)		1.03 (0.64-1.65)	
Cumulative duration (days)						
Never	1.00 (Reference)	0.88[d]	1.00 (Reference)	0.86[d]	1.00 (Reference)	0.86[d]
≤ 150	1.08 (0.68-1.73)		1.07 (0.67-1.72)		1.07 (0.67-1.72)	
> 150	0.98 (0.60-1.59)		0.97 (0.60-1.58)		0.97 (0.60-1.58)	
Average daily dose (DDDs)						
Never	1.00 (Reference)	0.87[d]	1.00 (Reference)	0.90[d]	1.00 (Reference)	0.90[d]
≤ 1	1.02 (0.68-1.54)		1.02 (0.68-1.53)		1.02 (0.67-1.53)	
> 1	1.02 (0.56-1.88)		1.01 (0.55-1.86)		1.01 (0.55-1.87)	
**Non-DiCCBs**						
Ever vs. never users	1.56 (0.99-2.46)	0.06	1.55 (0.98-2.44)	0.06	1.55 (0.98-2.44)	0.06
Cumulative dose (DDDs)						
Never	1.00 (Reference)	0.01[d]	1.00 (Reference)	0.02[d]	1.00 (Reference)	0.02[d]
≤ 94.5	1.11 (0.53-2.35)		1.11 (0.52-2.35)		1.10 (0.52-2.34)	
> 94.5	2.02 (1.15-3.53)		1.99 (1.14-3.49)		2.00 (1.14-3.50)	
Cumulative duration (days)						
Never	1.00 (Reference)	0.01[d]	1.00 (Reference)	0.02[d]	1.00 (Reference)	0.01[d]
≤ 106	1.10 (0.52-2.34)		1.10 (0.52-2.33)		1.10 (0.52-2.32)	
> 106	2.02 (1.16-3.54)		2.00 (1.14-3.50)		2.01 (1.15-3.51)	
Average daily dose (DDDs)						
Never	1.00 (Reference)	0.05[d]	1.00 (Reference)	0.05[d]	1.00 (Reference)	0.05[d]
≤ 1	1.56 (0.77-3.16)		1.55 (0.76-3.13)		1.55 (0.77-3.13)	
> 1	1.55 (0.86-2.76)		1.54 (0.86-2.75)		1.54 (0.86-2.74)	
Individual molecules[f]						
**Amlodipine (ever vs.never)**	1.07 (0.69-1.67)	0.75	1.07 (0.69-1.67)	0.76	1.07 (0.69-1.67)	0.76
**Lercanidipine (ever vs.never)**	1.28 (0.86-1.90)	0.23	1.27 (0.85-1.89)	0.24	1.27 (0.85-1.89)	0.24
**Verapamil (ever vs.never)**	1.19 (0.65-2.19)	0.57	1.18 (0.64-2.17)	0.59	1.19 (0.65-2.18)	0.58
**Diltiazem (ever vs.never)**	2.19 (1.19-4.03)	0.01	2.19 (1.19-4.03)	0.01	2.19 (1.19-4.03)	0.01
**Other CCBs[g] (ever vs.never)**	0.67 (0.38-1.21)	0.19	0.67 (0.37-1.20)	0.18	0.67 (0.37-1.20)	0.18

CCBs, calcium channel blocker; IR, incidence rate (per 1,000 person-years); HR, hazard ratio; CI, confidence interval; DDD, defined daily dose.

HRs, 95% CIs, and P-values were computed using Cox proportional hazards regression for time-varying variables with age as the timescale.

All models are adjusted for time-invariant variables (type of residence, education level, parity, age at first menarche, and caffeine consumption) and time-varying variables (smoking status, body mass index, physical activity level, menopausal status, number of consultations in the previous 6 months, type 2 diabetes, gout, cardiovascular diseases, hypertension, lipophilic statins, and LABA and ultra-LABA beta2 agonists).

[a] Additional adjustment for time-varying constipation.

[b] Additional adjustment for time-varying cardiac arrhythmia.

[c] Additional adjustment for time-varying constipation and cardiac arrhythmia.

[d] P-value for linear trend.

[e] Amlodipine, the other non-amlodpine DiCCBS (felodipine, isradipine, lacidipine, manidipine, nicardipine, nifedipine, nimodipine, and lercanidipine) and non-DiCCBs (bepridil, verapamil, and ditiazem) were included in the same mutually adjusted model.

[f] Amlodipine, lercanidipine, verapamil, ditiazem, and other calcium channel blockers (felodipine, isradipine, lacidipine, manidipine, nicardipine, nifedipine, nimodipine, bepridil) were included in the same mutually adjusted model.

[f] Felodipine, isradipine, lacidipine, manidipine, nicardipine, nifedipine, nimodipine, bepridil.

Table S12 | Association of CCBs use with the incidence of Parkinson's disease: analyses with a more specific version of the algorithm (specificity=sensitivity=90%).

	5-year lag (576 PD patients)		No lag (773 PD patients)	
	Model 3		Model 3	
**CCBs**	**HR(95% CI)**	**P- value**	**HR(95% CI)**	**P- value**
**CCBs**	1.20 (0.91-1.58)	0.20	1.18 (0.97-1.44)	0.09
**Mutually adjusted model A**				
**Amlodipine**	1.13 (0.73-1.77)	0.58	1.40 (1.08-1.82)	0.01
**Non-amlodipine DiCCBs**	1.04 (0.73-1.49)	0.82	1.04 (0.81-1.32)	0.78
**Non-DiCCBs**	1.64 (1.04-2.58)	0.03	1.39 (1.00-1.94)	0.05
**Mutually adjusted model B**				
**Amlodipine**	1.13 (0.72-1.76)	0.59	1.42 (1.09-1.84)	0.01
**Lercanidipine**	1.29 (0.86-1.93)	0.22	0.92 (0.68-1.23)	0.56
**Verapamil**	1.24 (0.68-2.29)	0.48	1.13 (0.73-1.74)	0.59
**Diltiazem**	2.29 (1.24-4.22)	0.01	1.70 (1.06-2.70)	0.03
**Others CCBs**[d]	0.70 (0.39-1.26)	0.24	1.11 (0.81-1.52)	0.52

CCBs, calcium channel blocker; DiCCBs, dihydropyridine calcium channel blocker; HR, hazard ratio; CI, confidence interval.

[a] HRs, 95% CIs, and P-values were computed using Cox proportional hazards regression for time-varying variables with age as the timescale. Amlodipine, non-amlodpine DiCCBs (felodipine, isradipine, lacidipine, manidipine, nicardipine, nifedipine, nimodipine, and lercanidipine) and non-DiCCBs (bepridil, verapamil, and ditiazem) were included in the same model (mutually adjusted model A). Amlodipine, lercanidipine, verapamil, ditiazem and other calcium channel blockers (felodipine, isradipine, lacidipine, manidipine, nicardipine, nifedipine, nimodipine, bepridil) were included in the same model (mutually adjusted model B).

[b] Model 1 further adjusted for time-invariant variables: type of residence, education level, parity, age at first menarche, and caffeine consumption, and for time-varying variables: smoking status, body mass index, physical activity level, menopausal status, number of consultations in the previous 6 months, type 2 diabetes and gout.

[c] Model 2 further adjusted for time-varying cardiovascular diseases, hypertension, lipophilic statins and beta2 agonists (LABA and ultra-LABA).

[d] Felodipine, isradipine, lacidipine, manidipine, nicardipine, nifedipine, nimodipine, bepridil.

Table S13 | Case-control studies on the association between use of calcium channel blockers and incidence of Parkinson's disease.

Author, year (country)	Data source	Number of participants (% women)	Origin of controls	Case recruitment period	Lag-time (years)	Exposure	Exposure definition	Outcome definition	Adjusted OR and 95% confidence interval	Exposure subgroup	Adjustment variables	Calculation of doses/durations
Ton et al., 2007 (US)[12]	Group Health Cooperative (GHC)	556 : 191 cases and 365 controls (NA)	GHC registrants without PD or other neurodegenerative disorders and matched on age, gender, duration of GHC registration and clinic	1992-2002	5	User vs. never user	GHC pharmacy database (at least 2 prescriptions)	Medical records reviewed by study neurologist (presence of at least 2 of the 4 cardinal signs)	CCBs: 0.85 [0.43-1.66]	No	Age, gender, smoking, length of GHC enrollment, clinical and β-blocker use	- Cumulative duration of use<br>- Cumulative standard doses<br>- Average daily standard dose
Becker[a] et al., 2008 (UK[13]	UK-based General Practice Research (GPRD)	7,274 : 3,637 cases and 3,637 controls (40%)	From database and matched on age, gender, general practice, index date and duration of history	1994-2005	-	Current use of ≥ 30 prescriptions for CCBs vs. non-CCBs users.	Drug prescriptions in the GPRD.<br><br>Current users = last prescription less than 90 days ago	Idiopathic PD code recorded for the first time during the study period and no previous diagnosis of drug-induced parkinsonian disorder. (Algorithm)	CCBs: 0.77 [0.63-0.95]<br>CCBs Women: 0.66 [0.47-0.93]<br>DiCCBs: 0.78 [0.62-0.99]<br>Non-DiCCBs: 0.76 [0.52-1.10]	- Dihydropyridine: nifedipine, nimodipine, felodipine, amlodipine, lercanidipine, nicardipine, isradipine, lacidipine<br>- Non-dihydropyridine: Vérapamil et diltiazem	BMI, smoking, cardiovascular, metabolic and psychiatric diseases and dementia, diuretics and statins.	No
Ritz et al., 2010 (Denmark) [14]	Nationwide prescription database and National Hospital Register	11,582 : 1,931 cases and 9,651 controls (41,9%)	Population register matched by year of birth and sex	2001-2006	2	User vs. never user	National database of prescriptions (2 prescriptions or more)	Hospital records: first inpatient or outpatient registration of PD or first prescription of PD drugs.	DiCCBs (without amlodipine): 0.73 [0.54-0.97]<br>Amlodipine: 1.04 [0.87-1.25]<br>Non-DiCCBs: 1.14 [0.93-1.39]	- Centrally acting dihydropyridine: Felodipine, isradipine, nicardipine, nifédipine, nimodipine, nitrendipine, lacidipine, lercanidipine<br>- Peripherally acting Dihydropyridine: Amlodipine	Age, gender, diagnosis of chronic obstructive pulmonary disorder and Charlson comorbidity score.	- Duration of use and intensity of use in relation to DDDs

PD, Parkinson's disease; CCBs, Calcium channel blocker; DiCCBs, dihydropyridine CCBs; Non-DiCCBs, non-dihydropyridine CCBs; NA, not available; DDD, defined daily dose.

[a]Sensitivity analysis: risk of a diagnosis of PD in association with the use of calcium antagonists in a subgroup of cases and controls without diagnosed hypertension (receiving calcium antagonists other than for hypertension): the estimated risk for current use of calcium antagonists was 0.60 [0.42-0.86].

Table S14 | Cohort studies on the association between use of calcium channel blockers and incidence of Parkinson's disease.

Study (Year)	Data source (country)	No. Of participants (% women)	Follow-up (mean, years)	Exposure definition	Exposure	Outcome definitin	Lag-time (years)	No. Of cases	Adjusted HR or RR (95% CI)	Analysis of specific CCBs	Dose-response analysis	Smoking adjustment	Dosage calculation	Model
Louis (2009)[15]	NEDICES (Spain)	3,942 (NA)	1994-1998 (NA)	Self-report	Fixed	Self-report + neurological examination / medical records<br><br>At least 2 cardinal signs	-	30	CCBs : 1.24 [0.43-3.60]	No	No	No	No	Yes vs. No
Simon (2010)[16]	NHS, HPFS (US)	171,355 (70)	1986-2002 (NA)	Self-report	Fixed	Self-report + medical records + verification by neurologist<br><br>Certain or probable case or 2 of the 3 cardinal signs	-	514	CCBs women : 0.81 [0.32-2.03]<br><br>CCBs : 1.18 [0.73-1.92]	No	No	Yes	No	Users (with or without other antihypertensive drugs) vs non-users
					TV				CCBs < 4 years : 1.06 [0.68-1.65]<br>CCBs ≥ 4 years : 1.05 [0.67-1.65]				No	Current users vs. Never users of CCBs
Pasternak (2012)[a 17]	Danish Civil Registration System, Prescription Drug Registry, National Patient Registry (Denmark)	2,573,281 (NA)	1998-2006 (NA)	At least 2 consecutive prescriptions	TV	Diagnosis in the National Patient Registry and at least one prescription for an antiparkinsonian drug	-	5,968	DiCCBs : 0.71 [0.60-0.82]<br>DiCCBs women : 0.67 [0.53-0.85]<br>Amlodipine : 0.70 [0.59-0.83]<br>Felodipine : 0.64 [0.40-1.02]<br>Nifedipine : 1.09 [0.59-2.03]<br>Non-DiCCBs : 0.64 [0.42- 0.96]	DiCCBs : amlodipine, felodipine, isradipine, lacidipine, lercanidipine, nicardipine, nifedipine, nilvadipine, nitrendipine.<br><br>Non-DiCCBs : verapamil and diltiazem.	Yes<br><br>High dose amlodipine defined as 10 mg/day and a standard dose as 5 mg/day. High dose felodipine defined as 10 mg/day and standard dose as 2.5 mg/day or 5 mg/day	No	No (DDD in Prescription Drug Registry)	New users vs. Non-users
							2	NA	DiCCBs : 0.99 [0.82-1.20]					
Lee (2014)[b 18]	NHIRD (Taïwan)	65,001 (54)	2005- NA (4.6)	Drug claims	TV	Diagnostic of PD by neurologist in hospital	-	NA	CCBs : 0.75 [0.59-0.96]<br>CCBs (Women) : 0.66 [0.47-0.93]<br>DiCCBs : 0.71 [0.57-0.90]<br>Non-DiCCBs : 0.73 [0.52-1.04]<br>Amlodipine : 0.83 [0.67-1.03]<br>Felodipine : 0.72 [0.54-0.95]<br>Nifedipine : 0.80 [0.61-1.06]<br>Lercanidipine : 0.63 [0.33-1.22]<br>Nitrendipine : 0.57 [0.28-1.14]<br>Lacidipine : 0.99 [0.41-2.38]<br>Central-acting CCBs: 0.69 [0.55-0.87]<br>Peripheral-acting CCBs: 0.84 [0.67-1.04]	DiCCBs : Amlodipine, Felodipine, Nifedipine, Lercanidipine, Nitrendipine, Lacidipine<br><br>Non-DiCCBs : NA<br><br>Central-acting CCBs: Felodipine, Nifedipine, Lercanidipine, Nitrendipine, Lacidipine<br><br>Peripheral-acting CCBs: Amlodipine, Verapamil and Diltiazem	Yes<br><br>Cumulative DDD Quartiles	No	DDD	Any use vs. Beta-blocker
							1.5	NA	CCBs : 0.86 [0.66-1.12]					

Study (Year)	Data source (country)	No. Of participants (% women)	Follow-up (mean, years)	Exposure definition	Exposure	Outcome definitin	Lag-time (years)	No. Of cases	Adjusted HR or RR (95% CI)	Analysis of specific CCBs	Dose-response analysis	Smoking adjustment	Dosage calculation	Model
Tseng (2021) [c 19]	NHIRD (Taïwan)	107,207 (NA)	2000-2013 (NA)	+ 90 DDD of DiCCBs	TV	More than 5 consultations with diagnosis of PD + treatment with antiparkinsonian drugs	-	1,782	DiCCBs : 0.50 [0.45-0.55]	DiCCBs	Cumulative DDD: 90-180, 180-360, 360-720, >720<br><br>$p^d < 0.001$	No	DDD	With DiCCBs vs. Without DiCCBs

NEDICES, The Neurological Disorders in Central Spain; NHIRD, National Health Insurance Research Dataset; NHS, Nurses' Health Study; HPFS, Health Professionals' Follow-Up Study; PD, Parkinson's disease; CCBs, Calcium channel blocker; DiCCBs, Dihydropyridine CCBs; Non-DiCCBs, Non-dihydropyridine CCBs; DDD, defined daily dose; TV, time-varying; NA, Not available.

[a] New user design.

[b] Hypertensive patients.

[c] Newly hypertensive patients.

[d] Cochran-Armitage trend test.

Figure S1 | Study design for the analysis of the relation of CCBs use with the incidence of Parkinson's disease with a 5y-lag.

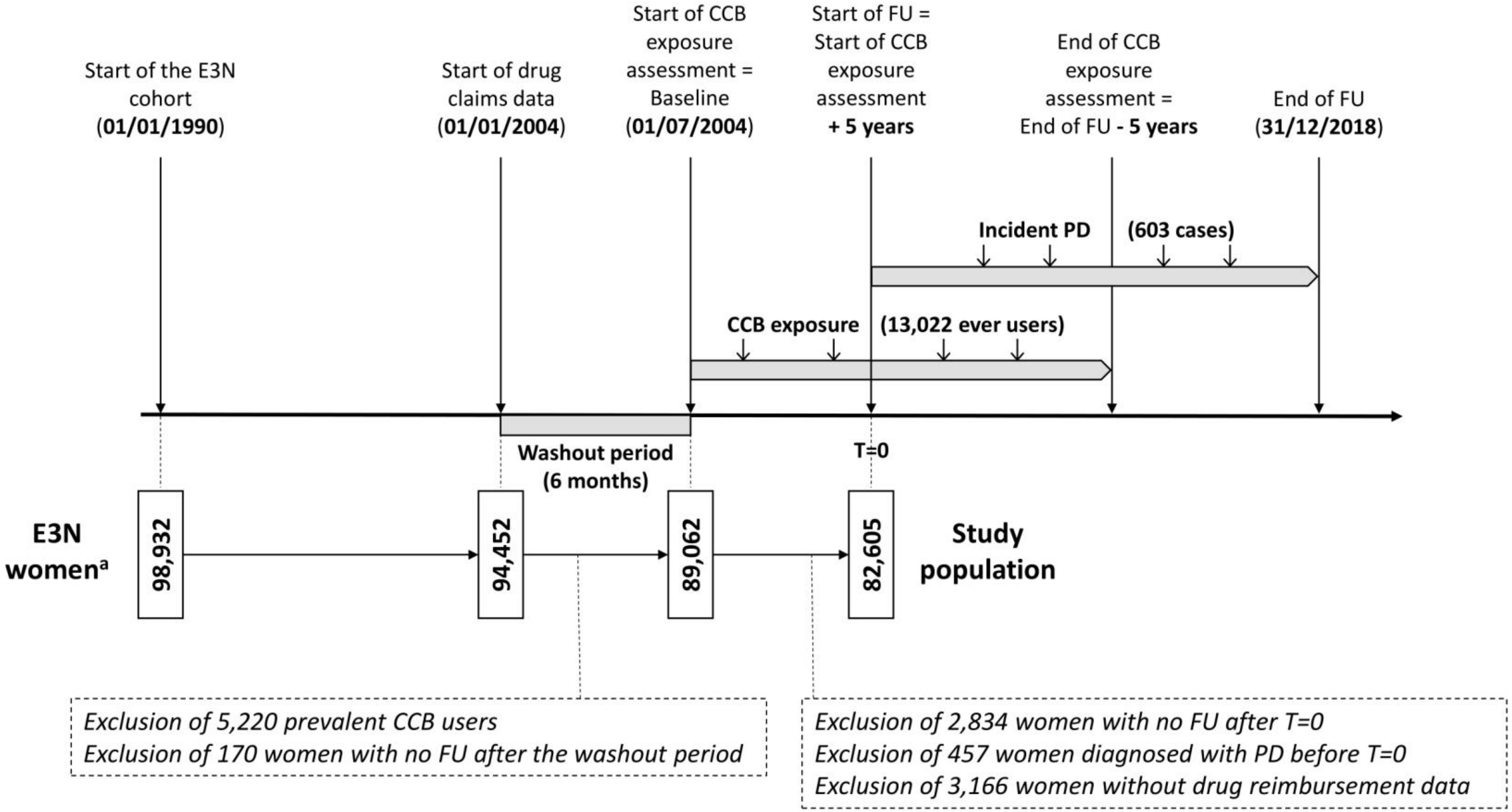

Abbreviations: CCBs = Calcium channel blocker; FU = follow-up; PD = Parkinson's disease.

[a]Of 98,995 E3N women recruited in 1990, we excluded 50 women with possible Parkinson's disease and 13 women with an unknown date of Parkinson's disease diagnosis.

Figure S2 | Study design for the analysis of the relation of CCBs use with the incidence of Parkinson's disease without an exposure lag.

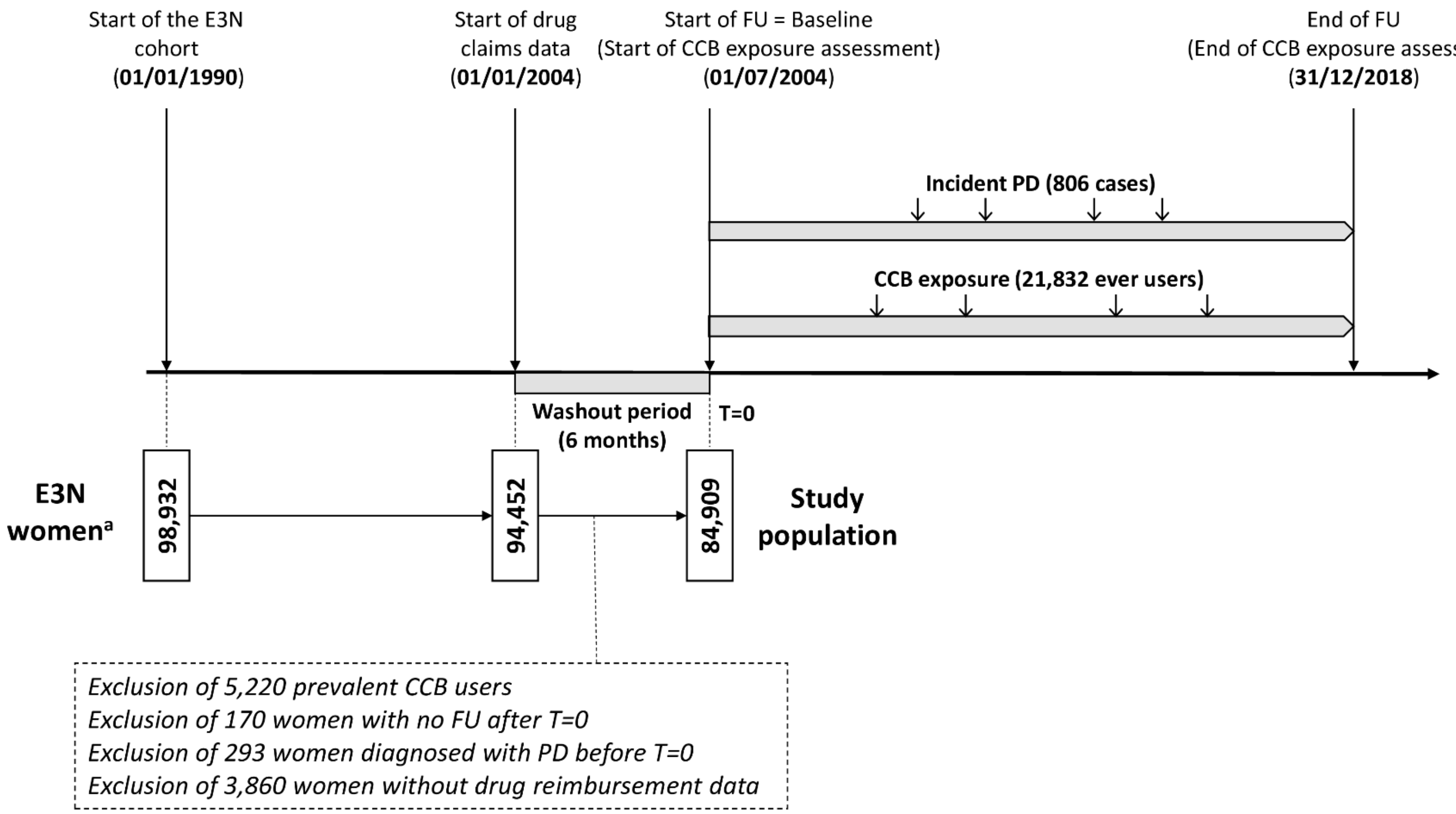

Abbreviations: CCBs = Calcium channel blocker; FU = follow-up; PD = Parkinson's disease.

[a]Of 98,995 E3N women recruited in 1990, we excluded 50 women with possible Parkinson's disease and 13 women with an unknown date of Parkinson's disease diagnosis.

Figure S3 | Directed acyclic graph showing the hypothesized causal structure of the variables included in the analyses.

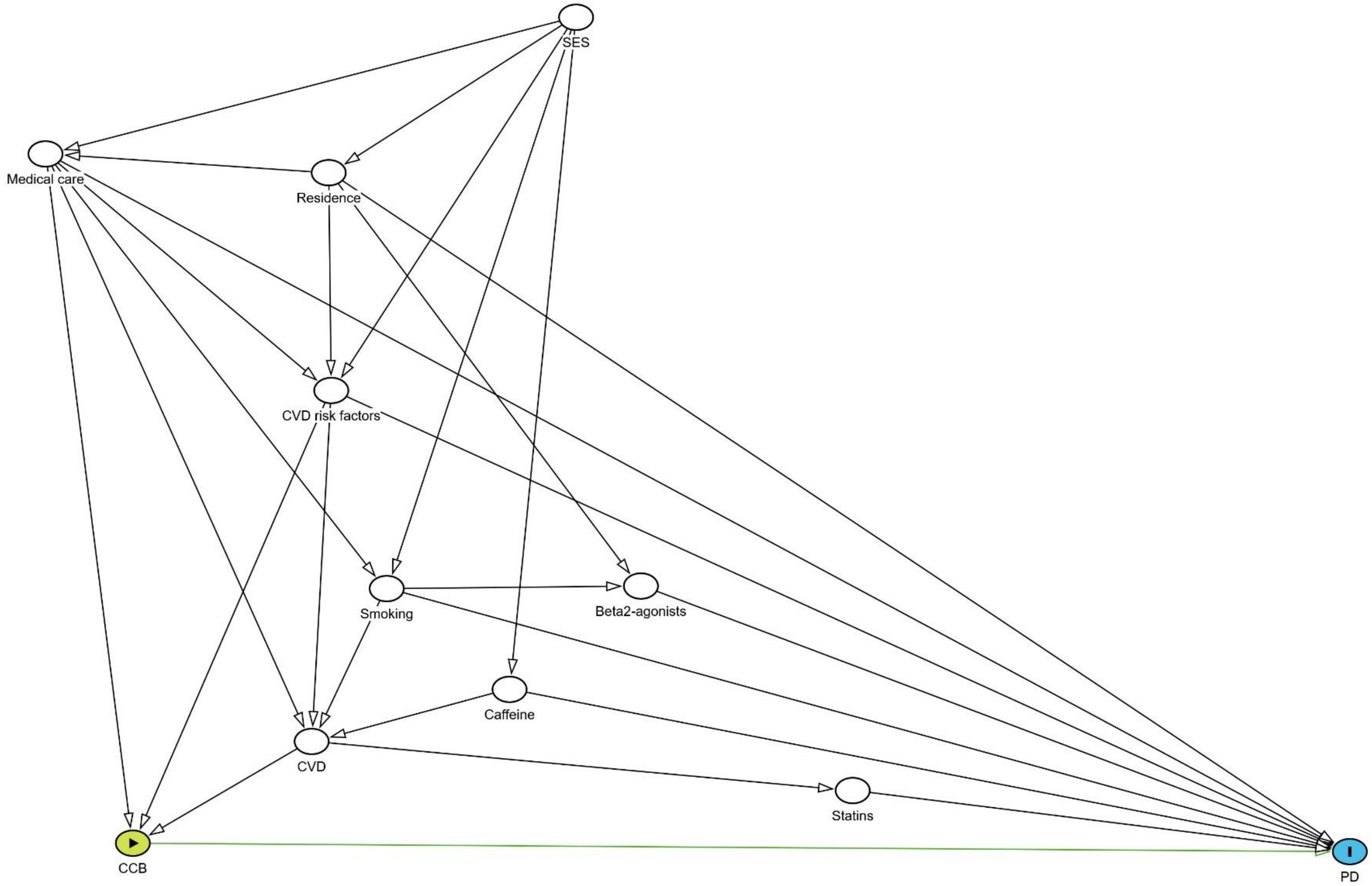

The DAG was drawn using DAGitty (https://dagitty.net/).

CCBs, calcium channel blocker; PD, Parkinson's disease; CV, cardiovascular disease; SES, socio-economic status.

CVD risk factors include reproductive characteristics (parity, age at first menarche), menopausal status, hypertension, body mass index, physical activity level, type 2 diabetes, and gout.

All the variables depicted with white circles represent confounders of the association between CCBs (exposure) and PD (outcome).

Supplementary references

1. Bellou V, Belbasis L, Tzoulaki I, Evangelou E, Ioannidis JP. Environmental risk factors and Parkinson's disease: An umbrella review of meta-analyses. *Parkinsonism Relat Disord* 2016;23:1-9;doi:10.1016/j.parkreldis.2015.12.008.

2. Portugal B, Artaud F, Domenighetti C, et al. Body Mass Index, Abdominal Adiposity, and Incidence of Parkinson Disease in French Women From the E3N Cohort Study. *Neurology* 2023;100(3):e324-e335;doi:10.1212/wnl.0000000000201468.

3. Portugal B, Artaud F, Degaey I, et al. Association of Physical Activity and Parkinson Disease in Women: Long-term Follow-up of the E3N Cohort Study. *Neurology* 2023;101(4):e386-e398;doi:10.1212/wnl.0000000000207424.

4. Chang H, Wang B, Shi Y, Zhu R. Dose-response meta-analysis on urate, gout, and the risk for Parkinson's disease. *NPJ Parkinsons Dis* 2022;8(1):160;doi:10.1038/s41531-022-00433-5.

5. Pesce G, Artaud F, Roze E, et al. Reproductive characteristics, use of exogenous hormones and Parkinson disease in women from the E3N study. *Brain* 2023;146(6):2535-2546;doi:10.1093/brain/awac440.

6. Frigerio R, Elbaz A, Sanft KR, et al. Education and occupations preceding Parkinson disease: a population-based case-control study. *Neurology* 2005;65(10):1575-1583

7. Liu W, Tang J. Association between diabetes mellitus and risk of Parkinson's disease: A prisma-compliant meta-analysis. *Brain Behav* 2021;11(8):e02082;doi:10.1002/brb3.2082.

8. Nabauer M, Gerth A, Limbourg T, et al. The Registry of the German Competence NETwork on Atrial Fibrillation: patient characteristics and initial management. *Europace* 2009;11(4):423-434;doi:10.1093/europace/eun369.

9. Nguyen TTH, Fournier A, Courtois E, et al. Statin Use and Incidence of Parkinson's Disease in Women from the French E3N Cohort Study. *Mov Disord* 2023;38(5):854-865;doi:10.1002/mds.29349.

10. Nguyen TTH, Fournier A, Courtois E, et al. Use of β-adrenoreceptor drugs and Parkinson's disease incidence in women from the French E3N cohort study. *J Park Dis* 2025;Accepted;doi:doi: 10.1177/1877718X251330993.

11. Gross A, Racette BA, Camacho-Soto A, Dube U, Searles Nielsen S. Use of medical care biases associations between Parkinson disease and other medical conditions. *Neurology* 2018;90(24):e2155-e2165;doi:10.1212/WNL.0000000000005678.

12. Ton TG, Heckbert SR, Longstreth WT, Jr., et al. Calcium channel blockers and beta-blockers in relation to Parkinson's disease. *Parkinsonism Relat Disord* 2007;13(3):165-169;doi:10.1016/j.parkreldis.2006.08.011.

13. Becker C, Jick SS, Meier CR. Use of antihypertensives and the risk of Parkinson disease. *Neurology* 2008;70(16 Pt 2):1438-1444;doi:10.1212/01.wnl.0000303818.38960.44.

14. Ritz B, Rhodes SL, Qian L, Schernhammer E, Olsen JH, Friis S. L-type calcium channel blockers and Parkinson disease in Denmark. *Ann Neurol* 2010;67(5):600-606;doi:10.1002/ana.21937.

15. Louis ED, Benito-Leon J, Bermejo-Pareja F, Neurological Disorders in Central Spain Study G. Antihypertensive agents and risk of Parkinson's disease, essential tremor and dementia: a population-based prospective study (NEDICES). *Neuroepidemiology* 2009;33(3):286-292;doi:10.1159/000235641.

16. Simon KC, Gao X, Chen H, Schwarzschild MA, Ascherio A. Calcium channel blocker use and risk of Parkinson's disease. *Mov Disord* 2010;25(12):1818-1822;doi:10.1002/mds.23191.

17. Pasternak B, Svanstrom H, Nielsen NM, Fugger L, Melbye M, Hviid A. Use of calcium channel blockers and Parkinson's disease. *Am J Epidemiol* 2012;175(7):627-635;doi:10.1093/aje/kwr362.

18. Lee YC, Lin CH, Wu RM, Lin JW, Chang CH, Lai MS. Antihypertensive agents and risk of Parkinson's disease: a nationwide cohort study. *PLoS One* 2014;9(6):e98961;doi:10.1371/journal.pone.0098961.

19. Tseng YF, Lin HC, Chao JC, Hsu CY, Lin HL. Calcium Channel blockers are associated with reduced risk of Parkinson's disease in patients with hypertension: A population-based retrospective cohort study. *J Neurol Sci* 2021;424:117412;doi:10.1016/j.jns.2021.117412.
